# Progress, opportunity, and perspective on exosome isolation - efforts for efficient exosome-based theranostics

**DOI:** 10.7150/thno.41580

**Published:** 2020-02-19

**Authors:** Dongbin Yang, Weihong Zhang, Huanyun Zhang, Fengqiu Zhang, Lanmei Chen, Lixia Ma, Leon M. Larcher, Suxiang Chen, Nan Liu, Qingxia Zhao, Phuong H.L. Tran, Changying Chen, Rakesh N Veedu, Tao Wang

**Affiliations:** 1 Department of Neurosurgery of Hebi People's Hospital; Hebi Neuroanatomical Laboratory, Hebi, 458030, China.; 2 School of Nursing, Zhengzhou University, Zhengzhou, 450001, China.; 3 Henan Key Laboratory of Ion-beam Bioengineering, Zhengzhou University, Zhengzhou, China, 450000.; 4 Guangdong Key Laboratory for Research and Development of Nature Drugs, School of Pharmacy, Guangdong Medical University, Zhanjiang 524023, China.; 5 School of Statistics, Henan University of Economics and Law, Zhengzhou 450046, China.; 6 Centre for Molecular Medicine and Innovative Therapeutics, Murdoch University, Perth 6150, Australia.; 7 General Practice Centre, Nanhai Hospital, Southern Medical University, 528244, Foshan, China.; 8 School of Medicine, Wake Forest University, Winston Salem, NC 27101, USA.; 9 School of Medicine, and Centre for Molecular and Medical Research, Deakin University, 3216, Australia.; 10 The First Affiliated Hospital of Zheng Zhou University, Zhengzhou 450001, China.; 11 Perron Institute for Neurological and Translational Science, Perth 6009, Australia

**Keywords:** Exosome, microvesicle, extracellular vesicle, microfluidic, diagnosis, separation

## Abstract

Exosomes are small extracellular vesicles with diameters of 30-150 nm. In both physiological and pathological conditions, nearly all types of cells can release exosomes, which play important roles in cell communication and epigenetic regulation by transporting crucial protein and genetic materials such as miRNA, mRNA, and DNA. Consequently, exosome-based disease diagnosis and therapeutic methods have been intensively investigated. However, as in any natural science field, the in-depth investigation of exosomes relies heavily on technological advances. Historically, the two main technical hindrances that have restricted the basic and applied researches of exosomes include, first, how to simplify the extraction and improve the yield of exosomes and, second, how to effectively distinguish exosomes from other extracellular vesicles, especially functional microvesicles. Over the past few decades, although a standardized exosome isolation method has still not become available, a number of techniques have been established through exploration of the biochemical and physicochemical features of exosomes. In this work, by comprehensively analyzing the progresses in exosome separation strategies, we provide a panoramic view of current exosome isolation techniques, providing perspectives toward the development of novel approaches for high-efficient exosome isolation from various types of biological matrices. In addition, from the perspective of exosome-based diagnosis and therapeutics, we emphasize the issue of quantitative exosome and microvesicle separation.

## 1. Exosomes and two obstacles for exosome-based basic and applied investigations

The physiological function of the human body relies on effective and precise cell communication 1. Apart from contact-dependent and soluble molecule-mediated signal transduction, we have expanded our knowledge of cell communication in recent years to include the role of exosomes as a new form of signaling system 2. Exosomes are small extracellular vesicles with diameters between 30-150 nm that feature a double-layer lipid membrane structure. Exosomes can be produced by almost all kinds of cells under both physiological and pathological conditions, and are widely distributed through easily accessible body fluids such as blood, saliva, breast milk or urine. The discovery of exosomes was first reported in 1987 3. For many years, exosomes were assumed to be “junk” produced during the maturation process of cells 3. However, with the recent isolation of various proteins, lipids and genetic materials (e.g., miRNA, mRNA, DNA molecules as well as long-noncoding RNAs) from different types of exosomes 4, their crucial roles in cell communication and epigenetic regulation have been recognized 2. Importantly, whether under pathological or physiological conditions, exosome contents are finely regulated by their parental cells to pass information from the parental cells to other cells for specific functions 5. In turn, the functional states of the parental cells can be estimated by analyzing their exosome contents 5, which lays the foundation for exosome-based diagnosis, especially non-invasive liquid biopsy. Apart from in disease diagnosis, exosome application also features in various biomedical fields including drug delivery 6, cell-free vaccine development 7, and regenerative medicine 8. Recently, the application of exosomes as a potent substitute for maternal cells in immunotherapy and regenerative medicine has been demonstrated with *in vivo* animal work, serving as the basis for several ongoing clinical studies 9. Indeed, exosomes hold high potential in the treatment of various diseases; by 2018 exosome-related investigations attracted $250 million (USD) in investments and are expected to exceed $1 billion (USD) by 2021 10. Accordingly, there are currently 127 exosome-related clinical trials being registered at Clinicaltrials.gov (versus 26 trails for the year of 2017) involving treatment and diagnosis of multiple types of diseases. Considering that the key discovery of genetic material in exosomes was not published until 2007 2, the speed of clinical translation of exosome-based theranostics has far exceeded the original expectations 9.

However, the general atmosphere around exosome-based clinical application is still pessimistic. As addressed by a recent position paper of the International Society for Extracellular Vesicle (ISEV) 9, the explosive attention and substantial capital investment in clinical translation of exosomes is mainly due to open intellectual property space, which provides incentive for early movers. Whether these efforts are successful depends on the solution of several key technical issues, as historically, there have been two main technical hindrances that restrict the basic and applied researches of exosomes 11. The first is how to simplify the exosome extraction procedure and improve the yield of exosomes; the second is how to effectively distinguish exosomes from other extracellular vesicles, especially from functional microvesicles.

In this work, by comprehensively analyzing existing exosome isolation techniques, we provide suggestions and insights for future exosome separation methods and related applications. In addition, from the perspective of exosome-based diagnosis and therapeutics, we also emphasize the issue of quantitative exosome and microvesicle separation.

## 2. Six major separation strategies exploring different physiochemical properties of exosomes

Exosomes are nano-sized extracellular vesicles distributed through vastly complex body fluids, which makes high-yield exosome isolation challenging 12. For instance, although ultracentrifugation has been the “gold standard” for exosome separation due to its high processing capacity, high levels of protein aggregate and lipoprotein contamination in exosome samples prepared through this method greatly compromises their quantification and functional analysis 13. Because a single method fitting a variety of sample sources is not practicable, efforts have been made to exploit different physiochemical and biochemical properties of exosomes. Until now, six classes of exosome separation strategies have been reported, including ultra-speed centrifugation, ultrafiltration, immunoaffinity capture, charge neutralization-based polymer precipitation, size-exclusion chromatograph, and microfluidic techniques, with unique sets of advantages and disadvantages for each technique (**Table 1**). In this section, by analyzing principles, procedures, and advantages and disadvantages of individual techniques, we provide a panoramic view of current exosome isolation strategies. This overview not only facilitates the optimization of exosome isolation strategies in different applications, but also provides new outlooks for the development of novel devices and approaches for efficient exosome isolation.

Importantly, as we will discuss in Section 3, although vesicles prepared by current approaches are commonly denoted as exosomes, it should be noted that the term “exosome” is often used improperly in published articles or clinical trials. Apart from exosomes, the “exosome samples” prepared via current techniques also include a great number of non-exosome vesicles such as microvesicles, apoptotic bodies and ectosomes 11, 14. This is because of their vast overlap in physicochemical properties and the currently limited knowledge about the molecular mechanisms of exosome biogenesis and release. Such non-exosome particles, especially functional microvesicles, compromise the accuracy and reliability of exosome-based theranostics. For this reason, the 2018 ISEV guideline position paper has suggested that due to the lack of pure exosome separation with current techniques, the commonly used term of exosome should be replaced with the more collective term of extracellular vesicle 15. As a result, unless specifically stated, the term “exosome” used in this article denotes a mixture of small extracellular vesicles such as exosomes, apoptotic bodies, microparticles, microvesicles, ectosomes, as well as oncosomes.

### 2. 1 Ultracentrifugation-the gold standard exosome isolation approach

With the capacity to generate centrifugal forces as high as 1,000,000 ×g (100,000-150,000 ×g is commonly used for exosome separation), ultracentrifugation is an optimal process for separating small particles including bacteria, viruses, and cellular organelles. As such, ultracentrifugation readily translates to exosome isolation and has contributed to many pioneering exosome explorations 3, 16. We will next discuss the application and main features of three common ultracentrifugation methods to demonstrate the details of this “gold standard” exosome isolation strategy.

#### 2.1.1 Differential ultracentrifugation contributed to most pioneering exosome studies

**Figure 1:**
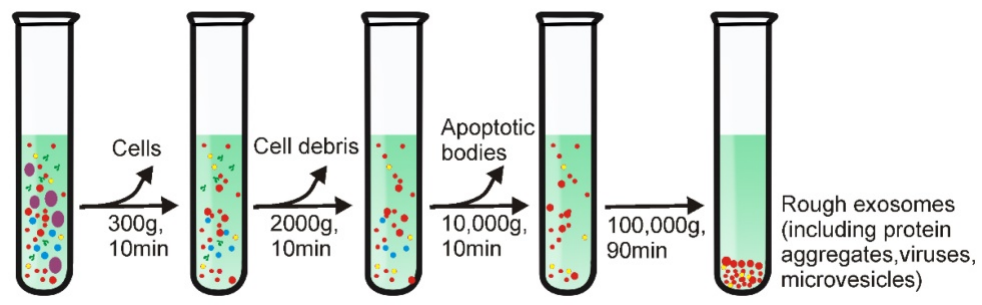
** Schematic representation of differential ultracentrifugation-based exosome isolation.** Differential ultracentrifugation is performed by multiple cycles of centrifugation with centrifugal forces from 300 ×g up to 100,000 ×g. After each centrifugation step, pellets including cells, cell debris as well as apoptotic bodies are removed while the supernatant was collected for further centrifugation. After the last centrifugation (i.e., 100,000 ×g), exosomes-containing pellets and contaminant proteins are collected by removing the supernatant. The centrifugation is performed at 4°C.

Differential ultracentrifugation, also referred to as simple ultracentrifugation or the pelleting method, is the most commonly reported strategy for exosome separation (45.7%) 17,18. The principle of differential ultracentrifugation is quite simple - under certain centrifugal forces, different extracellular components of a fluidic sample can be sequentially separated based on density, size as well as shape. This method was first reported by Johnston in 1987 to isolate exosomes from the culture medium of reticulocyte tissue 16. Later, in 2006, differential ultracentrifugation was further optimized by Thery and colleagues with a set of increasing centrifugal forces 19. As demonstrated in Figure 1, depending on the nature of the tested samples, a cleaning step may be first conducted to eliminate large bio-particles by low-speed centrifugation (e.g., 300 ×g), followed by multiple cycles of centrifugation with centrifugal force from 2000 ×g up to 100,000 ×g, to sequentially remove contaminants such as cell derbies, apoptotic bodies and protein aggregates for purified exosome isolation. Importantly, this method easily scales up for large scale exosome preparation. Although commonly used ultracentrifugation tubes have a relatively low volume capacity (~5-20 mL), existing liquid concentration devices (e.g., Centricon® Plus-70 Centrifugal Filter Units) 6 can facilitate the process of volumes of up to 200 mL with a 5-mL loading capacity ultracentrifugation tube.

Due to ease of use, little technical expertise requirement, and compatibility with large volume preparation without complicated sample pre-treatment, differential ultracentrifugation has been widely employed over the past 30 years to isolate exosomes from various sources such as cell culture medium, serum, saliva, urine, and cerebrospinal fluid 6, 20-22. However, it should be noted that extracellular fluids feature high heterogeneity. Under a certain centrifugal force, all components (including exosomes, microvesicles, and non-vesicles such as protein aggregates and lipoproteins) with buoyant density, size, and mass reaching a certain threshold can be precipitated at the bottom of the tube 23. Therefore, exosome samples prepared via differential ultracentrifugation often suffer from low purity, which potentially compromises many downstream applications, especially exosome-associated functional analysis 24. For example, in a well-designed comparison study, Paolini and colleagues used several different strategies to separate exosomes from the blood of patients suffering from multiple myeloma. In the subsequent functional study, they observed that exosomes prepared with differential ultracentrifugation (displayed high-amount contamination) demonstrated only poor and inconsistent biological functions compared to more purified exosome samples, which could induce prominent NF-κB nuclear translocation in endothelial cells 25.

Fortunately, to further improve the exosome isolation efficiency of this classical separation technique, various types of centrifugation strategies have been developed during the past two centuries through the exploration of the different physical properties of objects. Among these strategies, a widely used method is density-gradient centrifugation, which separates particles by density 26-28.

#### 2.1.2 Isopycnic & moving-zone density-gradient ultracentrifugation for high-quality exosome isolation

**Figure 2:**
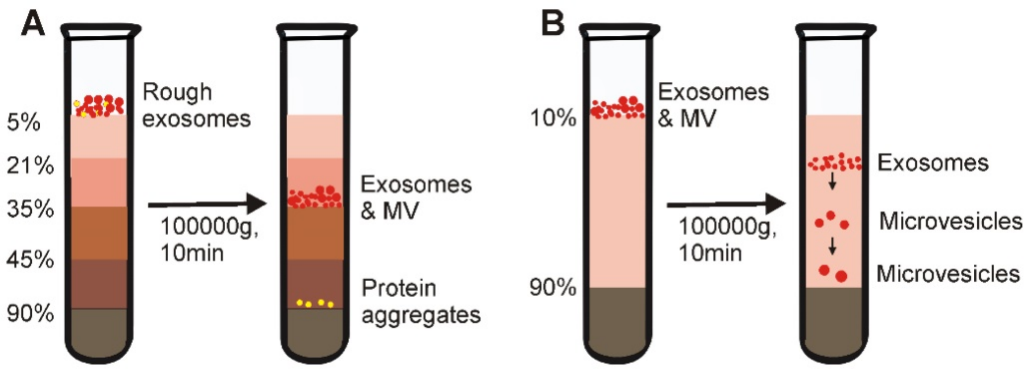
** Schematic representative of gradient density ultracentrifugation-based exosome isolation.** (A) Isopycnic density-gradient ultracentrifugation is prepared by adding medium in layers of progressively decreased density from bottom to top. After prolonged centrifugation, extracellular components including exosomes, apoptotic bodies and protein aggregates reach a static position in medium of similar density to each component. However, because isopycnic gradient ultracentrifugation depends solely on the density difference between different solutes in samples, this method cannot separate substances (e.g., microvesicles) with similar buoyant density to exosomes. (B) The moving-zone gradient ultracentrifugation normally consists two gradient medium sections. The top layer is a medium with density lower than all of the solutes of the sample. The bottom is a high-density cushion. As the density of the solutes are all greater than that of the gradient medium, after centrifugation, all solutes will be sequentially separated based on not only density, but also mass/size, thereby allowing the separation of vesicles of comparable density but varying size.

In 1937, Linderstorm-Lang discovered that after centrifugation in a density-gradient tube, objects of a particular density would remain suspended in medium of a similar density 29**.** Historically, the density-gradient-based centrifugation method has been commonly used in hematological study for the separation of subpopulations of blood cells, due to the differences in density of different cell types 30. Similarly, due to the density differences between different extracellular components, purified exosomes can be obtained through this method 31, 32. A typical density-gradient ultracentrifugation includes the following steps: First, layers of biocompatible medium with varying densities (e.g., iodoxinol or sucrose) covering the range of particle densities in the sample is placed into a tube, with gradually decreasing densities from bottom to top (Figure 2A). Next, the sample of interest is added onto the top of the density-gradient medium, followed by extended centrifugation for a prolonged period (e.g., 100, 000 ×g for 16 h) 6, 33. Eventually, the extracellular components, including exosomes, apoptotic bodies, and protein aggregates, gradually reach a static position (isopycnic position) in the layer of the same density. Through this method, components having different buoyant densities can be easily separated; protein aggregates concentrate at the bottom of the centrifugation tube while exosomes remain in the layer of medium between 1.10 and 1.18 g mL^-1^ 34. Again, in reference to Paolini and colleagues' comparison study 25, compared with differential ultracentrifugation and popular one-step precipitation kits (to be discussed in Section 2.4); density-gradient ultracentrifugation achieves the purest exosome samples for downstream applications. As a result, density-gradient ultracentrifugation has gained great popularity in recent years for exosome separation, representing around 11.6% of the currently used exosome strategies 17, 25.

However, such commonly used isopycnic ultracentrifugation depends entirely on the density difference between different solutes in samples. Although this method effectively separates exosomes from common contaminants such as protein aggregates, this method cannot separate extracellular vesicles with similar buoyant density (but different size) to exosomes (e.g., microvesicles) 35. To effectively address this technical issue, studies have used moving-zone density-gradient centrifugation (also termed as rate zonal centrifugation), which separates particles by both size and density 36. As shown in Figure 2B, the moving-zone ultracentrifugetion features a medium with a density lower than that of all solutes in the sample. As the density of the solutes is greater than that of the gradient medium, after centrifugation, all solutes in the sample will be sequentially separated based on not only density, but also mass/size, thereby allowing the isolation of vesicles with comparable densities but varying sizes (e.g., exosomes, viruses and large microvesicles) 36. However, unlike isopycnic ultracentrifugation, because the concentration of the medium in this type of ultracentrifugation is lower than that of all sample components, all insoluble particles can be pelleted at the bottom of the tube after prolonged centrifugation (hence why it is called moving-zone centrifugation). Consequently, the centrifugation time must be carefully determined for optimal exosome isolation. In order to minimize exosome pelleting, a high-density medium is normally loaded in the bottom of the centrifuge tube to serve as a cushion (Figure 2B).

Despite various advantages and wide application, ultracentrifugation does have its shortcomings. For instance, although gradient ultracentrifugation is capable of purifying exosomes with minimal contamination, the processing volume of this method is limited by the thin loading zone 13. Additionally, ultracentrifugation approaches require not only expensive equipment, but also highly trained technicians, especially for gradient ultracentrifugation. Furthermore, as has been emphasized by previous studies 37, the structure and biological function of the isolated exosomes can be detrimentally affected by prolonged periods of ultra-centrifugal force, which is very unfavorable for downstream applications such as exosome-based functional studies and drug development. In light of this issue, other size-based separation strategies such as ultrafiltration and size-exclusion chromatography have been introduced. As we discuss in the next sections, various simplified and highly efficient exosome separation kits based on such techniques are now commercially available.

### 2.2 Ultrafiltration holds potential for industrial scale exosome preparation

Similar to conventional filtration methods, ultrafiltration uses an ultrafine Nano-membrane with different MWCO (molecular weight cut-off) to isolate extracellular vesicles from clinical samples or cell culture medium and differentiate between exosomes and co-vesicles by size 38. Compared with the ultracentrifugation method, ultrafiltration-based exosome isolation dramatically shortens processing time and does not require special equipment, presenting an ideal substitute to the classical ultracentrifugation strategy 39. Importantly, by easily adjusting filter size, ultrafiltration allows researchers to sort specific subsets of small extracellular vesicles (including exosomes) with defined particle sizes 40.

**Figure 3:**
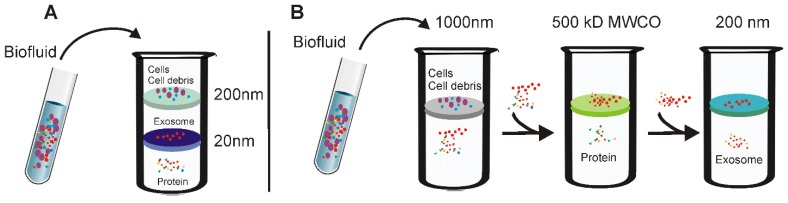
** Schematic demonstration of ultrafiltration-based exosome separation.** (A) Tandem- configured microfilter. Extracellular fluids are passed through tandem-configured microfilters with defined size-exclusion limits around 20-200 nm. When passing through the two membranes, large vesicles including cell debris, apoptotic bodies and the majority of microvesicles are trapped in the 200-nm membrane, while vesicles with diameter from 20 to 200 nm are retained on the lower 20 nm filter. (B) Sequential ultrafiltration. Extracellular fluids are first passed through a 1000-nm filter to get rid of larger particles (e.g., cells or cell debris); then the filtrate is passed through a second filter with 500-kD cut-off to remove small particles such as free proteins; finally, exosomes <200 nm are collected via a 200-nm filter.

Based on this principle, several simplified and easy-to-use ultrafiltration devices have been recently developed to facilitate the fast preparation of exosomes with yield comparable to that of the ultracentrifugation method 40. As demonstrated in Figure 3, there are two types of ultrafiltration devices that have been well-developed. The first is a tandem-configured microfilter (Figure 3A), which consists of two tandem-configured microfilters with defined size-exclusion limits around 20-200 nm 41. When passed through the two membranes, large vesicles including apoptotic bodies, as well as the majority of microvesicles; are trapped in the 200-nm membrane whilst vesicles of 20-200 nm diameter remain on the bottom and smaller particles such as proteins pass through the 20-nm microfilter. On the other hand, sequential ultrafiltration is another popular method for exosome isolation (Figure 3B) 42. In this mode, extracellular fluids are first passed through a 1000-nm filter to get rid of large particles including cell debris, cells, and apoptotic bodies. After that, the filtrate is then passed through a second filter with 500-kD MWCO to remove free proteins and other small particles. Finally, exosomes with diameters between 50-200 nm can be collected from the filtrate with a 200-nm filter. Based on this sequential ultrafiltration protocol, the Bio Scientific Corporation recently developed a kit called “ExoMir™ exosome isolation” 43. By leveraging a syringe filter-based adjustable fractionation process, this device enables large volume processing (10-25 mL/run), rapid isolation of small extracellular vesicles (including exosomes and microvesicles depending on filter size) from various types of fluids include serum, cerebrospinal fluid, and eukaryotic cell culture media. In addition, by including a second RNA isolation module, this kit allows real-time RNA isolation from the harvested small extracellular vesicles for further analysis.

**Figure 4:**
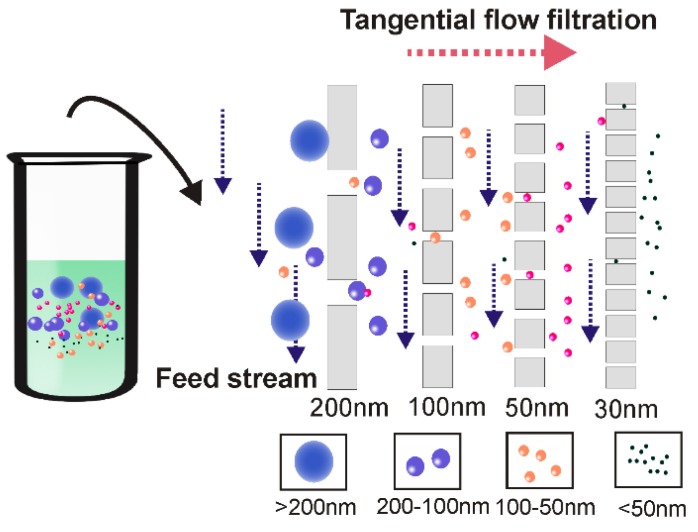
** Tangential flow filtration ensures highly efficient ultrafiltration.** During tangential flow filtration, the feed stream flows parallel to the membrane face. The applied pressure causes one portion of the flow stream to pass through the membrane according to the filter size. As the membrane is constantly under a parallel flow force, potential clogging can be efficiently minimized. During the tangential flow filtration procedure, the remainder is re-circulated back to the feed reservoir for repeated filtration, ensuring thorough filtration.

Over the past decade, due to high efficiency (minutes for ultrafiltration vs up to 16h for ultracentrifugation) and simplicity (does not require special equipment), ultrafiltration gained increasing popularity, representing around 5.4% of the currently used exosome isolation methods 17. However, this method has a few limitations. One of the most noticeable problems associated with the application of ultrafiltration is vesicle clogging and trapping, which potentially reduces the lifetime of the expensive membranes and leads to low separation yield 44, 45. Apart from pre-treatment with proteinase to reduce fluid sample viscosity 46, 47, tangential flow filtration techniques present an ideal solution to this problem 48. As demonstrated in Figure 4, during tangential flow filtration, the feed stream flows parallel to the membrane 49. Through manipulation of the hydrodynamic flow force, the pressure applied to the flow stream causes only part of the flow to cross the membrane. As the membrane is constantly under a parallel flow force, potential clogging can be efficiently minimized (via constant flushing). The remainder (retentate) can then be re-circulated back to the feed reservoir for repeated filtration during the tangential flow filtration procedure, thus allowing an automated procedure as well as high yield 50. Tangential flow filtration-based exosome preparation has been applied to separate exosomes for various clinical trials due to these advantages 38, 51, 52. In a recent clinical trial, dendritic cell-derived exosomes prepared by this method were able to effectively promote T-Cell response in a promising anti-cancer treatment 53. In 2017, the University of Texas MD Anderson Cancer Centre developed a notable three-step sequential filtration device designed for processing large volumes of bio-fluids based on tangential flow technique 40. The exosome isolation device consists of three separate modules where first, large particles such as cell debris are filtered out by a 100-nm filter which detains only inflexible solutes with sizes larger than 100 nm such as apoptosis bodies but allows flexible solutes to cross, even if they are larger than 100 nm in size. Next, tangential flow filtration is performed using 500-kDa MWCO hollow fibers to further deplete small contaminants such as proteins. Lastly, exosomes are isolated by filtering the retentate through a low-pressure filter with defined pore size (i.e., 100 nm). In addition to its fast processing, simplified procedure and isolation of exosomes with defined particle size, this semi-automated ultrafiltration strategy allows the isolation of extracellular vesicles on an industrial scale with minimum structural damage (maintaining functional integrity) via careful monitoring and maintenance of the transmembrane pressure, therefore holding great potential for exosome-based theranostic translations.

Apart from vesicle clogging and trapping, the co-presence of nanoparticles with sizes comparable to those of exosomes presents another limitation of ultrafiltration 54. The combination of two or more isolation methods (e.g., gradient ultracentrifugation) can address this problem 55, 56. Importantly, the transmembrane pressure applied during ultrafiltration, if performed improperly, could detrimentally affect the native state of the isolated exosomes, resulting in loss of function 42. For this reason, caution needs to be taken during the whole ultrafiltration procedure to avoid the collected exosomes from deformation and fragmentation 57, 58.

### 2.3 Size-exclusion chromatography allows separation of exosomes with minimal structural damage

**Figure 5:**
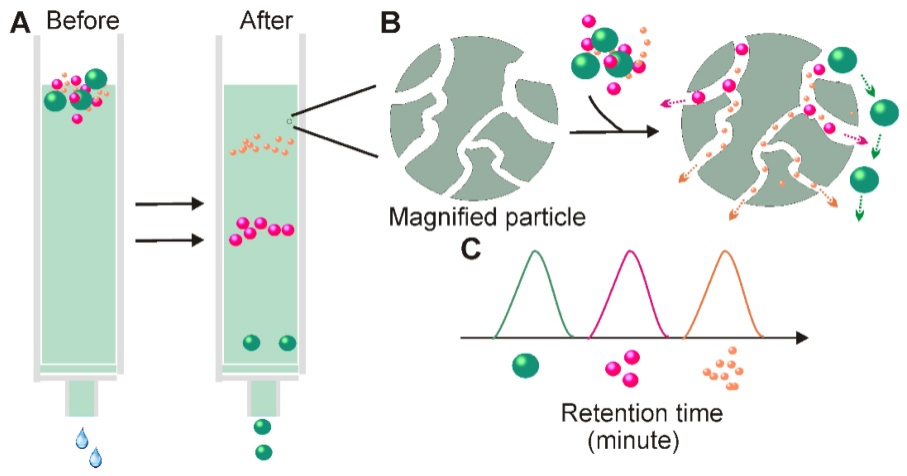
** Principle for Size-exclusion chromatography-based exosome isolation.** When passing a solution through a stationary phase consisting of porous resin particles, molecules can be separated according to size (A); While particles with hydrodynamic radii smaller than that of the pores of the stationary phase enter into the pores for longer traffic distance, larger particles, which cannot enter the pores move directly around the resin (B). This causes particles with different sizes to exhibit different retention times and therefore facilitate size-based separation.

In 1955, Grant H.L and Colin R.R invented a size-based separation technique termed size-exclusion chromatography (SEC) to isolate solutes of different molecular weights by passing aqueous solution through a column made of starch and water 59. When passing a liquid sample through a stationary phase consisting of porous particles, molecules with different hydrodynamic radii submitted to different fates. While molecules smaller than the pores of the stationary phase are slowed because they enter into the pores, larger molecules, which cannot enter the pores are forced around the porous particles and are eluted earlier from the column (Figure 5). Over the past 50 years, this method was dramatically improved through the introduction of various fine, porous materials such as dextran polymer (Sephadex), agarose (Sepharose), and polyacrylamide (Sephacryl or BioGel) 60. Long before the discovery of exosomes, SEC has been well-developed and widely applied to the high-resolution separation of large molecules or aggregates of macromolecules such as proteins, polymers, and various liposome particles 60-62. The knowledge acquired from SEC-based liposome isolation translates readily to exosome separation, as exosomes share many similar physical properties with liposomes. In merely 10 years of development, companies have developed various commercial SEC kits designed specifically for exosome isolation such as qEV (iZON) and PURE-EVs (Hansa Biomed).

In terms of exosome-based therapeutic application and functional studies, perhaps the most appealing feature of SEC is its ability to preserve the natural biological activities of the separated exosomes 63. Unlike ultracentrifugation and filtration, SEC is performed by passive gravity flow, which does not affect vesicle structure and integrity 58. The natural state of exosomes can be further enhanced by the selection of elution buffers with physiological osmolarity and viscosity (e.g., PBS) 64. Apart from maintaining exosome function, SEC has additional advantages. First, SEC requires minimal volumes. With commercially available SEC columns; volumes as small as 15 µL can be processed to achieve high-resolution, standardized, and reproducible exosome isolation suitable to exosome-based fingertip analysis 65. Second, SEC-based exosome collection is simple, compatible with various types of fluids, and an extra pre-treatment step is generally not required 14. Third, the SEC method saves time and labor. With selective porous materials and buffer systems, the whole process can be completed within a short and well-defined time period (e.g., 15 minutes) 66. Fourth, similarly to the ultrafiltration method, fine adjustment of the pore size of the applied materials can yield a defined subpopulation of extracellular vesicles 66. Lastly, compared to ultrafiltration-based separation, the contact-free manner of SEC (solutes do not interact with the stationary phases) ensures none or minimal sample loss and high yield 63. Given all these merits, it is not surprising that in recent years' SEC-based exosome isolation has becoming increasingly popular for exosome-based basic and clinical investigations. Importantly, this method is not only suitable for processing trace amount liquid samples, but also easily scalable and automated for high-throughput exosome preparation. Recently, iZON developed an automatic exosome isolation system (qEV Automatic Fraction Collector) based on the established SEC platform and weight-dependent segment and sample collecting systems which allows fast, precise, scalable and automated exosome isolation 67**.**

Despite various advantages, the SEC method also faces several challenges. According to a recent comparison study, exosomes prepared via SEC column commonly displayed wider size distribution, especially in the lower size range, suggesting the existence of contaminants with sizes similar to those of exosomes such as proteins aggregates and lipoproteins. To eliminate such contaminants, in the 2013 ISEV conference, Gardiner proposed an exosome isolation strategy by combining ultrafiltration and SEC 68. Later, the combined use of ultrafiltration and SEC was practiced in cell culture medium by Shu and colleagues 69. According to their assessment, in comparison with solely SEC or ultrafiltration, this combined strategy not only harvested exosomes with significantly improved purity, but also preserved exosome function. Similarly, functional exosomes were also prepared by Rood's group via combined application of ultracentrifugation and SEC 70.

### 2.4 High-yield polymer precipitation strategy coupling with issue of contamination

Along with the size-dependent exosome isolation strategies discussed above, polymer-induced precipitation presents another commonly used strategy for exosome isolation. Analogous to ethanol-mediated nucleic acid precipitation, highly hydrophilic polymers interact with water molecules surrounding the exosomes to create a hydrophobic micro-environment, resulting in exosome precipitation 46. Among various hydrophilic polymers, polyethylene glycol (PEG), a well-described, non-toxic polymer (a common excipient for pharmaceutical products) with the ability to remodel the water solubility of surrounding materials has been commonly used 71, and constitutes the foundation for several popular commercial exosome isolation kits, such as ExoPrep (HansaBioMed, Estonia), Total Exosome Isolation Reagent (Invitrogen, USA), ExoQuick (System Biosciences, USA), Exosome Purification Kit (Norgen Biotek, Canada), miRCURY as well as Exosome Isolation Kit (Exiqon, Denmark).

**Figure 6:**
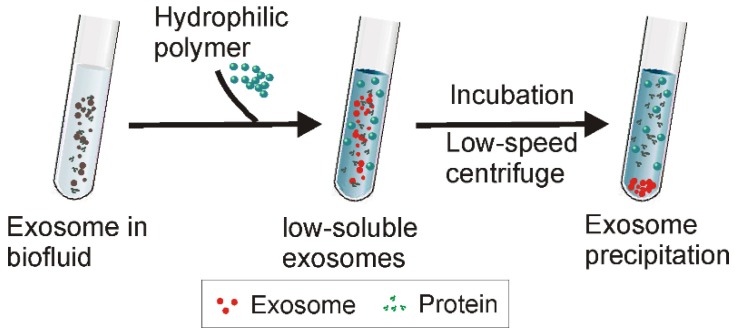
** Schematic of Polymer Precipitation Strategy.** After the addition of highly hydrophilic polymers to an exosome-containing solution, water molecules surrounding the exosomes are tied up by the polymers, lowering the solubility of the exosomes and inducing their subsequent precipitation. The exosomes can be easily collected with low-speed centrifugation.

Existing polymer-based exosome precipitation methods generally employ PEG with molecular weights from 6000 to 20000 Da 71. Firstly, a pre-treatment is required to remove big contaminant particles such as cell debris and apoptotic bodies, followed by incubation of the pre-treated samples with PEG solution at 4°C for overnight 72. Next, the precipitated exosomes are collected via low-speed centrifugation (1500 ×g) (Figure 6). With such a straightforward protocol, this method has been widely used to isolate exosomes from various types of samples such as blood, cell culture medium, cerebrospinal fluid, urine, and ascites 46, 73, 74. Since polymer precipitation methods do not require sophisticated equipment, this method is easily scalable to large preparation volumes with high yield. This method also allows fast disease diagnosis through the integration of various detection platforms for exosome (or protein/genetic material contents) analysis 75**.**

Typically, polymer precipitation-based exosome isolation is characterized by high yield. As shown in a recent comparison study using urinary samples, polymer precipitation achieved the highest yield of exosome and genetic contents (i.e., miRNAs and mRNAs) for subsequent profiling analysis, compared to differential ultracentrifugation and ultrafiltration methods 55. However, water-excluding polymers can precipitate not only exosomes, but also various water-soluble materials such as nucleic acids, lipoproteins, protein, and even viruses 76, 77, therefore the possibility of other extracellular contaminants could be very high. Indeed, after testing exosome samples collected via polymer precipitation through a mass spectrometry assay, noticeable detected protein contaminants included albumin and immunoglobulin, accompanied by residue polymer molecules 78, 79. Currently, although various techniques (e.g., Nanosight particle tracking analysis, vesicle flow cytometry, tunable resistive pulse sensing, electron microscopy, and surface plasmon resonance) have been developed for exosome quantification, a gold standard exosome quantification strategy has not been developed. All of these strategies have their limitations. For instance, although Nanosight Nanoparticle Tracking Analysis has been commonly employed, this method is expensive and restricted to a limited dynamic range for particle concentration measurements. In most current studies, exosome quantification relies on the measurement of the total protein content of the tested samples 6, 24. For this reason, polymer precipitation inevitably causes false quantification of exosome preparations due to the existence of nonspecific protein contaminations (as well as protein from other non-exosome particles) 80. In addition, the existence of such contaminants may also impair downstream analysis. In a recent comparison study, when Girijesh and colleagues treated human pancreatic cancer MiaPaCa cells with exosomes prepared via different methods, it was found that exosomes prepared by precipitation rather than other strategies resulted in unexpected cell toxicity 55. To further improve the polymer-based exosome preparation, apart from applying extra pre-clean (i.e., centrifugation) and post-clean (i.e., via sephadex G-25 column) steps or the combined application of two or more techniques 71, the recently reported aqueous two-phase (layer) system (ATPSs) presents another option 81.

**Figure 7:**
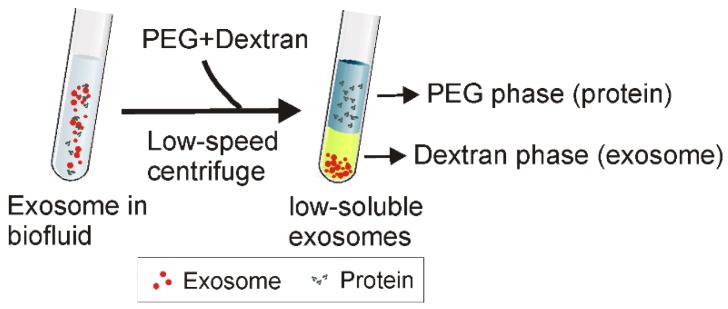
** Schematic of aqueous two-phase system-based exosome isolation.** When the more hydrophobic polyethylene glycol (PEG) and more hydrophilic dextran solutions are mixed, a two-phase system could occur. After addition of PEG and dextran to exosome-containing solutions followed by incubation and low-speed centrifugation, proteins and other big molecular complexes preferentially accumulate into PEG while exosomes preferentially accumulate into the dextran phase.

ATPS has been widely used to separate various substances including cells, proteins, and metal ions 82. As shown in Figure 7, the principle of ATPS is very similar to that of the traditional organic-water solvent extraction system. When the relatively more hydrophobic solution (e.g., PEG) and more hydrophilic and denser solution (e.g., Dextran) are mixed together, a two-phase system occurs, where PEG consists of the upper phase while dextran forms the lower phase. Accordingly, after adding PEG and dextran to exosome-containing solutions, followed by a low-speed centrifugation, particles with different physicochemical features separate into different phases. While exosomes preferentially accumulate in the dextran phase, proteins and other macromolecular complexes preferentially accumulate into the PEG phase. As reported, ordinary laboratory equipment and a mere 15-min incubation with the ATPS method yielded ~70% exosome recovery efficiency, about four times higher than the classical ultracentrifugation method 17. Despite the observation on subsequent PCR of the adverse effect of high biopolymer concentration and high solution viscosity (e.g., up to 1.5% dextran) 17, this method presents a promising, inexpensive and rapid exosome isolation strategy to simplify exosome-based various applications.

### 2.5 Immunoaffinity capture enables isolation of highly purified exosomes for *in situ* detection

**Figure 8:**
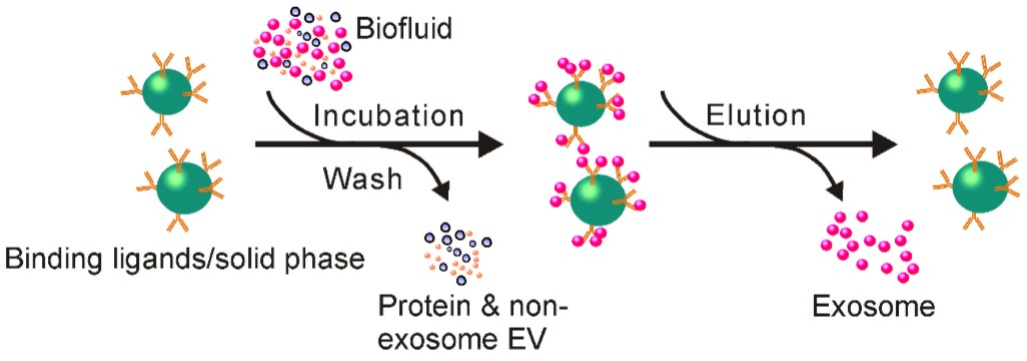
** Schematic of immunoaffinity-based exosome isolation.** First, antibodies recognizing exosome-specific markers are immobilized onto solid matrices. After incubating exosome-containing fluids with antibody-conjugated solid matrices, exosomes can be enriched onto such solid matrices. Free exosomes can be collected via an additional elution step.

The observation that some proteins and receptors that are common in all exosomes, regardless of their origin 83, provides an opportunity to develop immunoaffinity-based exosome isolation via the binding specificity between such protein markers and their corresponding antibodies (or exosome receptors and their ligands) (Figure 8). Theoretically, any protein or cell membrane components solely or highly presented on the membrane of exosomes and lacking solvable counterparts in the extracellular fluids could be used for immunoaffinity-based exosome capture. During the past few decades, various exosome markers have been recorded including lysosome associated membrane protein-2B, transmembrane proteins, heat shock proteins, platelet-derived growth factor receptors, fusion proteins (e.g., flotillins, annexins, and GTPases), lipid-related proteins, as well as phospholipases 84-87. Among them, transmembrane proteins such as Rab5, CD81, CD63, CD9, CD82, annexin, and Alix have been extensively exploited for selective exosome isolation 88, 89, resulting in several popular exosome isolation products including the Exosome isolation and analysis kit (Abcam), Exosome-human CD63 isolation reagent (Thermofisher) and Exosome Isolation Kit CD81/CD63 (Miltenyi Biotec). Remarkably, via specific biomarkers, immunoaffinity capture represents an ideal platform for isolating defined subpopulations of exosomes with specific origins. As demonstrated by a previous investigation, an EpCAM (overexpressed on tumor derived exosomes) antibody-coated magnetic bead system allowed the specific isolation of tumor-originated exosomes from not only cell culture medium but also various types of clinical samples 90. Recently, immunoaffinity separation systems designed for the isolation of specific subpopulation of exosomes have become commercially available (e.g., Exosome-Human EpCAM Isolation Reagent, Thermofisher). Obviously, collecting exosomes of specific origin not only facilitates the study of their parental cells, but also provides important indicators for disease diagnosis (for example, via detecting EpCAM positive exosomes to assess the existence of EpCAM related cancers).

#### 2.5.1 Solid matrices for antibody immobilization

For effective immunoaffinity-based exosome isolation, antibodies need to be fixed on a solid surface for exosome separation. Over the past few years, matrices including chromatography, beads, plates, and various types of microfluidic apparatuses have been used 13. Among these, submicron-sized magnetic particles (Figure 8), widely used for immuno-precipitation of recombinant proteins, have been most commonly used. This method not only yields high capture efficiency and sensitivity from its large surface and near-homogeneous processes, but also accommodates large starting sample volumes, therefore allowing upscaling or downscaling for specific applications 13. Moreover, as reported, this method could be directly transformed to a diagnostic platform through the detection of disease-specific markers (e.g., EpCAM, CD133, EGFR for cancer cells) on the isolated exosomes, facilitated by disease-specific antibody and magnetically activated cell sorting 91.

Plates and microchips are also popular matrices on which to develop immunoaffinity-based exosome separation systems, in addition to the commonly used magnetic beads. For example, by using microplate, an anti-CD9 antibody-based system has been devised to capture and quantify exosomes from various types of mediums such as urine and blood 92. Compared to the traditional ultracentrifugation method, this microplate-based immunoaffinity capturing device performs more efficiently in exosome isolation 92. Requiring only 400 µL of initial plasma sample in a one-hour procedure, this method isolates a comparable amount of exosome RNAs to that obtained by ultracentrifugation of 2.5 mL of plasma in a 16-hour procedure 92. Despite multiple disadvantages such as low volume processing capacity and relatively lower capture efficiency, this microplate-based method is suitable for the development of plate reader-based real-time diagnostic devices, especially for trace amount sample analysis 92.

#### 2.5.2 How to maintain the native state of exosomes? A major concern for immunoaffinity-based exosome isolation

Even though immunoaffinity-based exosome isolation ensures high-purity exosome isolation with an easy procedure, the non-neutral pH and non-physiological elution buffers (to separate exosomes from antibodies) associated with this method could irreversibly affect the biological function of the collected exosomes. The denatured exosome samples, although generally acceptable for diagnosis purposes (via assessing genetic and protein contents of exosome), are not favorable for exosome-based functional studies and various therapeutic applications 89, 93. Great efforts have been made to prepare exosome samples with intact structures. In an ingenious study, rather than using antibodies, Nakai and colleagues designed an exosome isolation device using the Ca^2+^-dependent Tim4 protein, which specifically binds to phosphatidylserine, a protein highly expressed on the exosome surface 94. By immobilizing Tim4 proteins onto magnetic beads, exosomes with high phosphatidylserine expression can be specifically isolated. Importantly, since the binding between Tim4 and exosomes is strictly dependent on Ca^2+^ 95, exosomes can be easily separated from Tim4-coated beads by the removal of Ca^2+^ through the addition of elution buffers containing Ca^2+^ chelators such as EDTA. Under such a gentle Ca^2+^ chelator treatment, the natural state of exosomes can be preserved. In another case, researchers from the Korea Advanced Institute of Science and Technology developed an Exosome-specific Dual-patterned Immune-filtration chip for specific exosome capture by introducing a chemically cleavable linker 3,3'-Dithiobis(sulfosuccinimidylpropionate) (DTSSP) between the antibody (anti-CD63) and the solid immobilization surface 96. With this method, a simple reduction step via tris(2-carboxyethyl) phosphine (TCEP) or Dithiothreitol (DTT) cleaves the antibody link to release the exosomes for reliable downstream analyses and applications. Such cleavable link-based antibody immobilization methods would be equally eligible to be applied to other immobilization matrices such as magnetic beads or plates for functional exosome isolation.

#### 2.5.3 How to isolate total exosomes rather than specific exosome groups? Another issue for immunoaffinity-based exosome isolation

Although immunoaffinity allows separation of a specific subpopulation of exosomes, at the same time it raises concerns about isolation of only the specific populations of exosomes that possess the antibody-recognized proteins. Considering the vast number of heterogeneous properties of exosomes in body fluids, this would result in an analytical bias (underestimations and false negatives) 97. This is especially true in cancer diagnosis, where protein expression undergoes constant modulation with the stage of cancer progress 98. In addition, specific isolation of only a subset of exosomes, although with higher purity, results in lower overall yield 39**.**

Taking this into account, apart from protein markers, other substances universally expressed on exosome membrane have been targeted, such as the saccharide chains (e.g., N-linked glycans, alpha-2,6 sialic acid, mannose, and polylactosamine) overexpressed on exosomal membranes 99. In a recent exploration, Samsonov and colleagues efficiently isolated exosomes from urine samples via lectin, a type of sugar-binding protein displaying high affinity to saccharide residues. The composition of these exosomes was further confirmed via miRNA profiling to be bulk exosomes rather than any particular exosome subpopulation 100. Furthermore, heparin, a type of highly sulfated glycosaminoglycan, also holds potential for total exosome isolation with its ability to non-specifically bind to a variety of proteins. As demonstrated in a recent study 101, heparin-affinity beads are capable of harvesting bulk exosomes from not only cell culture media but also human plasma, following an ultrafiltration step to remove free proteins.

#### 2.5.4 Chemical antibody-based next generation immunoaffinity approach

**Figure 9:**
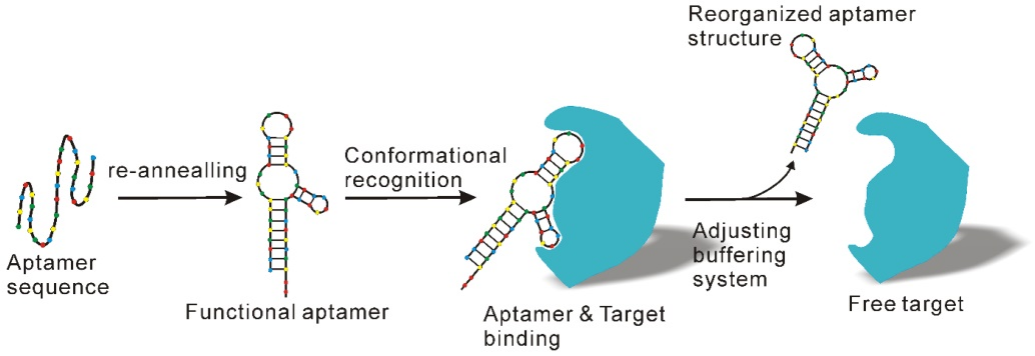
** Aptamer-mediated immunoaffinity.** Aptamers recognize and bind their target via conformational complementary. After adjusting key factors of the buffering system such as salt types and ionic strength, the shape of the aptamer undergoes change and releases the bound target molecules.

Even though antibody products possess distinct advantages, the high costs related to antibody development and production as well as their perishability significantly compromises their application, especially for large scale exosome preparation. To counteract these problems, apart from combined application with other methods as previously suggested 102**,** another option is to employ cheaper and more stable antibody substitutes, such as aptamer technologies. Aptamers, which are short single-stranded DNA or RNA sequences, can specifically recognize and bind to their targets with high affinity and specificity in a manner similar to antibodies 103, 104. However, unlike traditional antibodies, aptamers can be produced by *in vitro* chemical synthesis and exhibit several advantages such as low batch-to-batch variation, easy scaling up and down for different applications, extended shelf life, low or no immunogenicity, low production cost and easy chemical modification to improve binding properties 105, 106. Over the past years, several aptamer-mediated exosome isolation platforms have been developed 107, 108. Importantly, in addition to presenting a practicable option for immunoaffinity-based exosome isolation, aptamers also allow the preparation of natural exosomes with relatively little effort. As known, the recognition of aptamers and their target is strictly determined by tertiary structure 109, 110 (Figure 9), which in turn is determined by various factors such as temperature, ionic strength, as well as buffering systems. By adjusting the salt species and key ions (e.g., Mg^2+^ and K^2+^) to the formation of specific three-dimensional structure of aptamers, the binding capacity of aptamers can be easily remodulated under mild conditions 109, thereby releasing the captured exosomes with native structure and intact biological function.

### 2.6 Integrated microfluidic technique facilitates combinatorial exosome isolation and analysis

**Figure 10:**
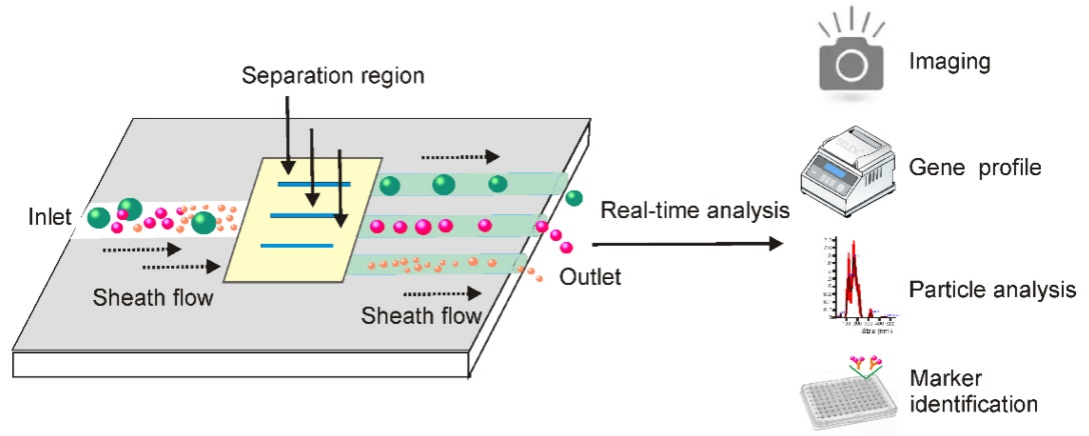
** Integrated microfluidic technique allows combined exosome isolation and analysis.** After adding exosome-containing fluids into the sheath medium, particles in the fluids including exosomes can be separated by different approaches based on the physical and biochemical properties of extracellular vesicles. Importantly, these miniaturized microfluidic apparatuses, facilitated by signal detecting platforms, allow for not only fast exosome isolation from small amount of body fluids, but also real-time exosome characterization for *in situ* diagnosis.

By exploring both the physiochemical and biochemical features of exosomes at microscale, the dramatic advances in microfabrication technologies have offered a valuable opportunity to develop lab-on-a-chip-type microfluidic systems for efficient exosome isolation 111-113. Facilitated by existing signal detecting platforms, these miniaturized microfluidic apparatuses allow for not only fast exosome isolation from fingertip amount of body fluids, but also real-time exosome characterization for *in situ* diagnosis (Figure 10). Indeed, microfluidic techniques are dramatically changing the landscape of exosome-based diagnosis by transferring the traditional two-step procedure (exosome isolation and characterization) to an integrated one-step process 113. This is especially valuable for non-invasive disease detection, such as early-stage cancer screening 114, 115.

#### 2.6.1 Immunoaffinity-based microfluidics

During the past decade, various forms of microfluidics have been invented through the exploration of different physiochemical properties of exosomes. Among them, the immuno-microfluidic technique has been most commonly used, resulting in commercial microfluidic products (e.g., ExoChip 116). Identical to the commonly used immunoaffinity-based exosome isolation method, the concept behind the immuno-microfluidic-based exosome separation devices involves the specific recognition of exosome markers by corresponding antibodies immobilized on the chips. In 2010, Chen et al. pioneered a microfluidic immunoaffinity apparatus for quick exosome isolation through the use of an anti-CD63 antibody 117. The resulting device was able to efficiently isolate exosomes from as small as 10 µL of cell culture medium and serum. Furthermore, by passing 300 µL of lysis buffer through the exosome-captured microchannel followed by air flushing, the group could easily obtain total RNAs from the captured exosomes. Subsequent tests demonstrated that significantly higher amounts of RNA could be collected via this chip system than by directly extracting RNAs from an equal amount of serum 117. Since then, high interest has been called toward either improving the efficiency or the specificity of such microfluidic-mediated exosome isolation systems.

##### 2.6.1.1 Efforts for immuno-microfluidic-based high-efficient exosome isolation

For a certain channel volume, larger binding surface area means more antibody immobilization and therefore higher exosome isolation efficiency. With this in mind, in 2016, Zhang and colleagues developed a microfluidic system that featured a graphene oxide/polydopamine (GO/PDA) nanointerface 118. The unique features of the GO-induced three-dimensional nano-porous structure provided a higher amount of surface area for efficient antibody immobilization and exosome capturing. As demonstrated, the developed CD81 antibody-microfluidic system not only greatly improved the efficacy of exosome isolation, but also the purity of the resulting exosome samples. Importantly, by encapsulating an ultrasensitive ELISA assay with both universal exosome biomarkers (CD81 and CD9) and cancer-specific biomarkers (EpCAM), this device allowed ultrasensitive *in situ* ovarian cancer detection in merely 2 µL of plasma 118. In another case, to increase the capture efficiency of an anti-CD9 antibody-based immuno-microfluidic chip, Hisey et al. introduced the “herringbone groove” (previously used to facilitate nanoparticle separation 119) pattern on the ceiling of the microfluidic channels. As expected, this novel design ensured significantly increased total surface area for antibody immobilization, and greatly improved exosome yield.

##### 2.6.1.2 Efforts for immuno-microfluidic-based highly specific exosome isolation

Nonspecific binding is a big issue for microfluidic-based immunoaffinity isolation as the method is incompatible with extra blocking and washing steps. This is different than conventional bead- or plate- based immunoaffinity approaches, where nonspecific binding between non-exosome vesicles and the exosome-specific antibodies (as well as the nonspecific binding between vesicles and the immobilization matrices (e.g., bead or plate surface)) can be efficiently eliminated via stringent blocking and washing. In recent years, the advances in nanotechnology have provided valuable opportunities to address this problem. For instance, Ramanathan et al. presented a powerful microfluidic system for high-specific exosome capturing and analysis, facilitated by the tunable alternating current electrohydrodynamic (ac-EHD) mediated nanoscale lateral fluid flow (also known as nano-shearing fluid flow) technique 120. As tested with three different antibodies, including anti-prostate specific antigen antibody, anti-CD9 antibody, and anti-human epidermal growth factor receptor 2 (HER2) antibody, this technology enabled efficient elimination of nonspecific/weak bound nanoparticles from the immune-affinity sites. Consequently, a greater than three-time increase in the sensitivity of exosome detection was recorded compared to that of traditional lateral flow assay 120. In another example, via employing inertial lift forces to effectively and rapidly exchange the washing solution around the exosome-antibody binding sites, Dudani et.al developed a microfluidic chip featured by a “spin-wash” procedure. A high signal-to-noise exosome isolation was achieved according to subsequent assessment 121.

As discussed in Section 1.5, although immunoaffinity-based exosome isolation allows easy exosome isolation, this method is limited by isolation of only specific subset of exosomes, high cost, and difficulty in maintaining the natural structure of exosomes. Size-dependent microfluidic isolation and contact-free separation strategies are two types of the most successful instances to address this problem. Next, we will discuss these two types of instruments to demonstrate recently developed examples of microfluidic devices.

#### 2.6.2 Size-based microfluidic separation techniques facilitate high-quality exosome isolation

**Figure 11:**
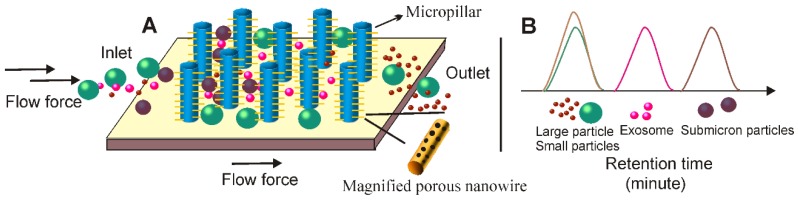
** Principle of the nanowire-based exosome trip system.** (A) Similar to SEC-based separation, a nanowire-on-micropillar hierarchy structure could be created via imprinting of porous silicon-consisting nanowires on the walls of the evenly separated micropillars. After adding exosome-containing fluids to the nanowire-on-micro-pillar tiered structure, particles in fluids are subject to different fates: (1). Larger particles (e.g., cell) are directly excluded from the sub-micrometer micropillar array; (2). Particles with submicron sizes (e.g., cell debris) are able to enter the micropillar interval but are unable to enter the 30-200 nm nanowire interval; (3). Small molecules (e.g., proteins) move across the nanowire interval without being obstructed; (4). Particles of 30-200 nm (e.g., exosomes) are arrested by the nanowire forest. (B) Particles with different sizes present different retention time and therefore facilitates size-dependent separation.

The first size-dependent microfluidic system discussed is the well-documented Exosome Total Isolation Chip (ExoTIC) 122. First, up to 10-mL solutions were filtered through a 0.22 µm nano-porous filter using a syringe pumper. Then, through the same syringe pumper and inlet, PBS was applied through the nano-porous filter to thoroughly clean and recover small extracellular vesicles (including exosomes) in a small volume (e.g., 200 µL). In this way, ExoTIC can effectively separate exosomes from both cell culture media and various types of body fluids such as lung bronchoalveolar lavage fluid, plasma and urine with limited effect on the native structure of exosomes. Importantly, this system was able to isolate exosomes from very small sample volumes (10-100 μL) with the yield around 4-1000 times greater than that of ultracentrifugation 122, ideal for point-of-care clinical testing. The second example is the nanowire-based exosome trip system (Figure 11). As demonstrated in Wang's study 123, following a similar principle with SEC, this device is characterized by nanowires (made of porous silicon) imprinted on the sidewalls of evenly separated micropillars to form a nanowire-on-micropillar hierarchy structure. According to the design, the interval between the nanowires can be adjusted from 30 to 200 nm to physically trap small extracellular vesicles, while the sub-micrometer micropillars, apart from offering support for nanowire anchoring, are effective for removing larger non-exosome particles such as cell debris and apoptotic bodies. Furthermore, the exosome isolation capacity of this device can be further enhanced by pre-loading exosome-specific antibodies onto the porous silicon nanowire to explore the immunoaffinity-based isolation. As tested, this microfluidic device can effectively isolate 40-100 nm exosome vesicles with a recovery rate of 60%, while allowing smaller (e.g., proteins) and large particles (e.g., cell, cell debris) to pass by unhindered. Importantly, via simply incubating in PBS buffer for 10 min, the chemical etching of the nanowire surface could be dissolved, thereby releasing the intact and purified exosomes for subsequent applications.

#### 2.6.3 Contact-free microfluidics-versatile tools for future exosome preparation

In addition to antibody and size-dependent microfluidics, rapid developments in microfabrication technology have enabled researchers to explore contact-free particle sorting mechanisms (e.g., elastic lift force, acoustic, and dielectrophoresis), for efficient, scalable, and high-quality exosome isolation.

**Figure 12:**
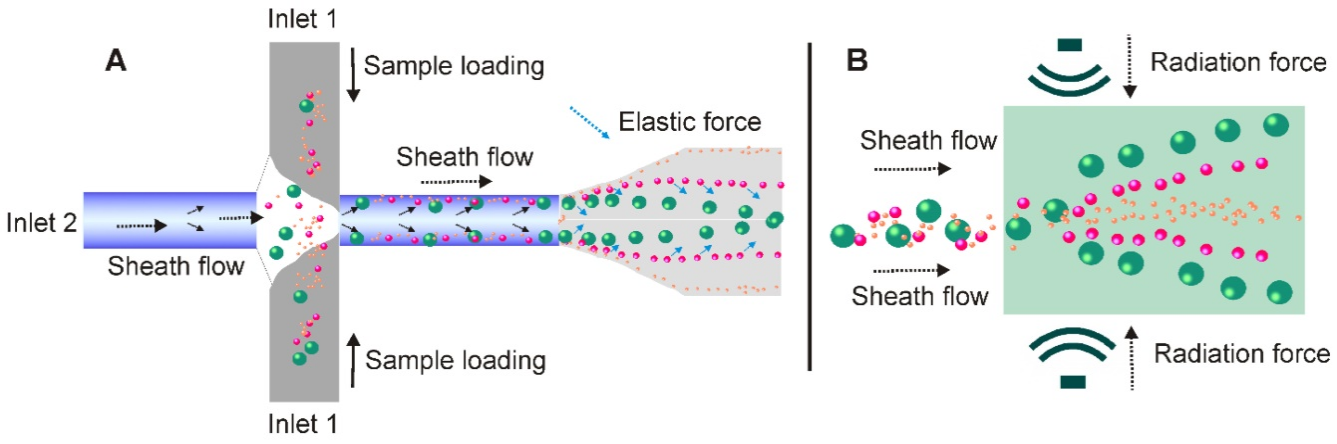
** Contact-free microfluidic enables simplified exosome separation procedure.** (A) In the viscoelastic medium flow-based microfluidic system, the exosome-containing fluids (added from inlet 1) meet the sheath flow (added from inlet 2) and are first aligned along the microchannel wall. After exertion of the elastic lift force that arises from viscoelasticity of the fluid, exosomes, and other extracellular components are driven toward the centreline of the microchannel according to their sizes, with larger particles eventually reach the centreline. (B) Under the pressure of ultrasound waves, particles with different mechanical properties (e.g., compressibility, size and density) experience differential radiation forces and results in contact-free and size-dependent exosome separation in a continuous manner.

In recent years, the unique migration pattern of particles in non-Newtonian viscoelastic fluids has attracted great interests. As documented, the elastic lift force created by a viscoelastic medium flow was able to control and manipulate the position of particles in a size-dependent manner (Figure 12A). Indeed, over the past few years, various viscoelastic flow-based microfluidic systems have been reported to isolate particles ranging from cancer cells, blood cells, bacteria, droplets to microspheres 124-126. In 2017, a contact-free viscoelastic microfluidic device was developed for size-dependent, continuous, and label-free exosome separation via manipulation of the viscoelastic force applied on exosomes by sheath fluid consisting of low concentrated (0.1%) biocompatible poly-(oxyethylene) (PEO) 125. After systematically optimizing key factors such as medium elasticity, microchannel geometry, and flow speed, this device allowed greater than 80% recovery rate and 90% purity, which is much higher than the 5%-25% recovery rate for ultracentrifugation. Although a size cut-off of 200 nm was demonstrated in this work, according to the authors, extracellular vesicles of defined sizes could be easily obtained by adjusting PEO concentration. Amazingly, with the capacity to process samples down to 100 μL in a mere 0.1s exosome passage time 125, this system holds potential to be used as a platform to separate exosomes from diverse biological samples for various types of theranostic applications. Importantly, without a sophisticated microfabrication structure or external force field, the contact-free feature of this viscoelastic exosome separation system can be continuously performed, significantly streamlining the design and produce of microfluidic-based exosome separation systems 127.

Under the pressure of ultrasonic waves, particles with different mechanical properties (e.g., compressibility, size and density) experience differential radiation forces 128. Based on this principle, in 2015, Lee and colleagues invented an acoustic nano-filter system allowing contact-free and size-specific exosome separation in a continuous manner 129. As demonstrated in Figure 12B, under ultrasound standing waves, the larger the particle is, the stronger radiation forces it will exert, and therefore display faster migration toward the pressure nodes, resulting in the separation of extracellular vesicles with defined particle sizes. When erythrocytes and cell culture medium were tested, such size-dependent acoustic technique could efficiently isolate purified exosomes 129. Importantly, such acoustic-based device allowed real-time control of the “size cut-off” via *in situ* electronic manipulation, which facilitated the isolation of exosomes with preferred sizes 129. In another case, Wu and colleagues reported a point-of-care device was capable of isolating exosomes from non-pre-treated raw blood samples in an automatic manner via the integration of microfluidic and acoustic techniques. This contact-free device provided the possibility to isolate intact, functional exosomes with high yield and purity for exosome-related therapeutics, disease diagnostics as well as health monitoring 130.

In addition to elastic lift force and acoustic force, the simplicity of electroactive strategies (without using instrumentation and specialized reagents) had also been explored for developing contact-free exosome isolation microfluidic systems 131. For instance, Davies et al. designed a microfluidic device that was able to effectively drive exosomes through a membrane while filter out other extracellular vesicles via electrophoresis within a microchannel, 132. To further improve electrode-based electrophoresis, dielectrophoresis had been employed by generating nonuniform electric fields through inserting insulating posts into the microchannel. After introduction to this nonuniform electric field, particles with different radii are subject to differing dielectrophoresis forces (inversely related to their radius) via polarization effects. Thus, under this electric field, smaller particles can be captured by greater gradients of the squared electric field (vice versa) and achieve size-dependent nanoparticle separation 133. In 2018, Shi et al developed a device based on such a mechanism 133. As reported, this microfluidic system could efficiently trap small extracellular vesicles near a glass nanopipette tip under 10 V/cm current 133. In another case, glioblastoma-originated exosomes were successfully isolated from human plasma in less than 30 minutes 134. These works were further improved in an effort led by Marczak for simultaneous isolation and concentration of exosomes 135. First, the authors developed a transverse local electric field by applying an ion-selective membrane. Under this electric field, the exosomes in a microfluidic chip could be easily forced out of the cross flow. After directing the exosome samples to agarose gels to eliminate undesirable cell debris, purified exosomes with defined particle size could be trapped and concentrated by ion-selective membrane. When tested with cell culture media and serum, this device was able to consistently capture between 60% and 80% of exosomes, as assessed by both nanoparticle tracking analysis and fluorescence spectroscopy 135. Importantly, with a concentration factor up to 15 ×, this device ensured efficient and reliable downstream exosome characterization 135. However, in spite of recent progress, additional investigations are still needed to further improve the efficacy and reliability of this electroactive strategy, especially for the optimal current (alternative current or direct current) and biological conditions for different samples.

#### 2.6.4 Microfluidic devices facilitate real-time exosome analysis

Although the application of exosomes has been used in diverse therapeutic purposes such as drug delivery, novel cell-free vaccine development and regenerative medicine, there has been an emphasis on their potential for disease diagnosis, especially in non-invasive cancer liquid biopsy 91. According to our statistics, diagnosis represents nearly half (54 in 127) of the currently registered exosome-related clinical trials (via Clinicaltrials.com). Apart from improving separation schedules to isolate high-quality exosomes with high yield, the establishment of simple and efficient detection techniques represents another major task in the development of microfluidic-based exosome separation devices. Indeed, in terms of exosome-based diagnostic applications, microfluidic devices possess multiple advantages for the development of low cost, reliable, real-time diagnostic devices to process fingertip amounts of easily attainable liquid samples such as serum, urine, breast milk, and saliva.

To facilitate post-separation exosome imaging, Ashcroft and colleagues produced a novel immuno-microfluidic device featuring a mica channel surface 136. Compare to commonly used glass or polymer materials, this antibody-bound mica surface, with a distinct atomically flat and hydrophilic surface, could be easily separated from the Polydimethylsiloxane (PDMS) fabricated flow cell base. This unique design thereby allows the attached exosomes and the mica surfaces to be directly imaged via ultrahigh-resolution atomic force microscopy. In another case, He and colleagues introduced an immuno-microchip integrated with ELISA assay as a method of quantitative detection. Unlike the traditional immunoaffinity-mediated exosome separation strategy, this approach allows the direct quantification of both surface and intra-vesicular markers of circular exosomes from 30 μL of plasma sample within 100 min 137. Later, a simplified continuous-flow microfluidic system named ExoSearch was developed 138. Facilitated by CD9 antibody (for exosome capture), CA-125 (for ELISA detection), EpCAM (for ELISA detection), and CD24 antibodies (for ELISA detection), this platform enabled rapid exosome isolation and *in situ* non-invasive cancer detection 138. Later, an anti-CD63 antibody-based device named ExoChip became clinically available 116. After isolation, exosomes collected by ExoChip were stained using a fluorescent carbocyanine dye (DiO) prior to plate reader-based quantification. Notably, ExoChip has been employed as a valuable exosome-mediated diagnostic system for various disease screening as it allows fast exosomal miRNA profiling 116. In addition to developing miniaturized devices for potable detection, more sophisticated detection platforms have also been integrated with current microfluidic systems for advanced applications. For example, Ueda and colleagues constructed a simplified microtip device enabling rapid and automated exosome isolation from various body fluids via conjugation of CD9 antibodies with highly porous monolithic silica microtips 139. By further combining this microtip device with a proteome-wide LC/MS/MS platform, the group established an exosomal biomarker discovery system that could simultaneously analyse up to 12 different samples. Through this system, the group was able to identify a specific antigen of lung cancer-derived exosomes, CD91 139.

It should be noted that the analytical sensitivity of reported “real-time on-chip exosome analysis” (including detection limit and response time) primarily depends on the specificity and binding capacity of the selected antibodies (for ELISA assay) as well as the sensitivity and compatibility of the utilized equipment. Therefore, such features of the analytical module need to be carefully investigated when designing real-time exosome analysis microfluidic devices.

## 3. Efficient exosome/microvesicle separation is critical for exosome and microvesicle-related investigations

**Figure 13:**
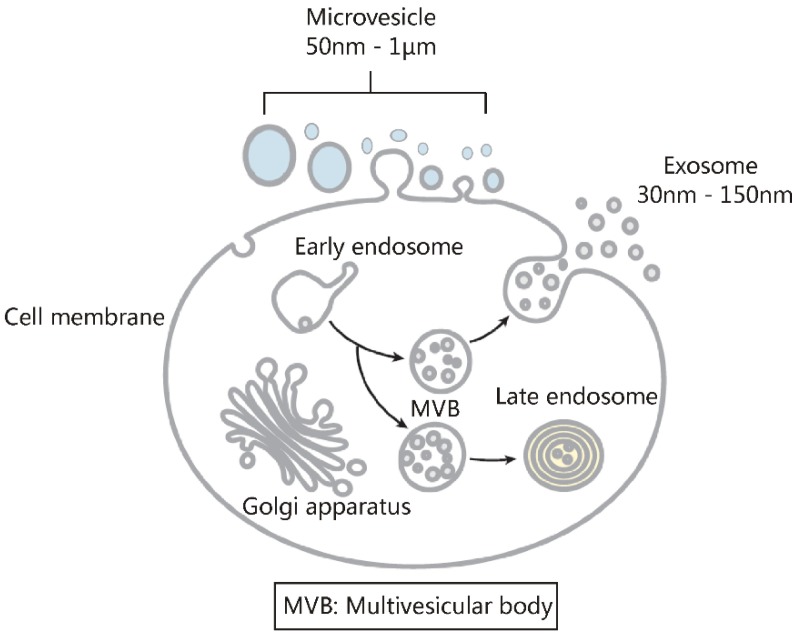
** Extracellular vesicles consist of mainly two types of vesicles with similar physiochemical properties.** Extracellular vesicles include exosomes and microvesicles. The main differences between them lie in their subcellular origins. Microvesicles are 50-1000 nm shedding particles from cell membrane; exosomes are 30-150-nm extracellular vesicles originated from endosomes, they are secreted into body fluids through exocytosis after cell membrane and multivesicular body fusion. Due to a lack of effective strategy to separate microvesicle and exosome, it is still difficult to precisely assess their physiochemical properties and functions.

As discussed in Section 1, the basic and applied researches of exosomes have been obstructed mainly by two issues 11. One is how to simplify the extraction procedure and improve the exosome yield; the other is how to effectively distinguish exosomes from other extracellular vesicles. In recent years, although standardized exosome extraction and qualitative/quantitative protocols are still not available, the rapid development in separation technology has in a large extent solved the problem of exosome isolation. For example, in order to obtain a sufficient amount of exosomes from cerebrospinal fluid for proteomics and nucleic acid quantification studies, researchers previously needed to collect 200-500 mL of cerebrospinal fluid to meet the requirements of ultracentrifugation 140. Nowadays, with newly developed exosome separation techniques such as immunoaffinity, chromatography and polymer precipitation, 6 mL of cerebrospinal fluid samples is sufficient to meet quantitative requirements 141. Improvements in the traditional polymer-based precipitation method have also addressed the long-standing obstacle of hydrophobic protein interference in urine exosome isolation 54. Today, with commercial exosome isolation kits and commonly available molecular biology equipment, exosomes can be extracted from trace amounts of clinical samples for subsequent studies in a short period of time, which greatly facilitates the basic and applied exosome studies. However, the second technical problem - how to effectively distinguish exosomes from other extracellular vesicles, still presents a major issue in exosome-related applications. We only have to consider the concept of the exosome to get an appreciation of what this means. The exosome was first proposed in 1987 3, denoting an extracellular vesicle originating from endosomes. It should be noted however that the concept of exosome is often not used properly in published articles or even clinical trials. As shown in Figure 13, apart from the endosome-originated exosomes, extracellular vesicles also contain a large number of microvesicles shed by the cell membrane. Unfortunately, due to their similar physicochemical properties and a large overlap in particle sizes, effective exosome/microvesicle separation still presents a very difficult task 11, 14. Instead of being inert materials as previously assumed, growing evidence is suggesting that microvesicles also display important biological functions 142, 143, although many of the published observations on “exosomes” actually describe the combined effects of exosomes and microvesicles. Given our limited knowledge of the biofunction of microvesicles, the existence of microvesicles in the tested exosome samples inevitably affects the exosome-based basic and applied studies in an unpredictable manner. As demonstrated by several recent odd findings, even for the same cancer cell type, “exosomes” collected by different groups could display quantitative or even qualitative differences in biological functions (either tumor promotion or inhibition) 2. As suggested, the different proportion of exosomes and microvesicles in the tested “exosome” samples may be the primary culprit of such controversial phenomena. For more accurate and reliable exosome-based diagnosis and therapeutic applications, an efficient exosome/microvesicle separation is necessary.

### 3.1 Solely relying on high-specific exosome markers is not sufficient for purified exosome isolation

Extracellular vesicles consist of mainly microvesicles and exosomes. In theory, the concentration of any component in the extracellular vesicle can be achieved by the isolation of another component. In practice, efforts aiming at efficient “exosome and microvesicle separation” have relied mainly on obtaining purified exosomes via identifying highly specific exosome biomarkers. This is due to (1) the lack of understanding of microvesicles and (2) the great potential of exosomes displayed in both basic research and theranostic applications in recent years. Theoretically, this strategy is feasible as the exosome proteins (including nucleic acids) are not a random combination of cell fragments but are integrated by a strict protein sorting mechanism to maintain their stable protein expression 2. Although current knowledge cannot describe in detail this particular sorting mechanism, the existence of such a mechanism itself provides a basis for the search for exosome-specific markers. As mentioned previously, during the past decades, various exosome biomarkers such as TSG101, CD81, CD9, CD63, CD37, CD82, CHMP2A, ALIX, RAB11B, CHMP4B, RAB11A, and RAB5 have been tested for immunoaffinity-based exosome Isolation. However, according to experience collected over the past 50 years, whether normal cells, stem cells or tumor cells, 100% specific markers do not exist 144; even classic exosome markers like CD63 and CD81 show expression on other subcellular organs 88 or even microvesicles 145, 146). Taking a step back, if the specificity of the employed exosome marker is not 100% specific (such as the currently used CD81 or CD63), then the proportion of microvesicles in the residue would not be calculated. Furthermore, under this circumstance it is even impossible to estimate the proportion of the exosome component.

In the face of this reality, we believe it is necessary for all scientists engaged in extracellular vesicle-related work to consider the following questions: (1) Is it a mistake to rely solely on the identification of novel exosome markers for exosome and microvesicle separation? (2) Are 100% purified exosomes and microvesicle components necessary for current basic and clinical research? (3) What is the key speed-limiting factor for current exosome studies?

### 3.2 Impurification is not the real problem for exosome-related studies

Is isolating 100% purified exosomes necessary for current exosome studies? In fact, the key obstacle facing current exosome studies is not the impurity of exosome samples, but the lack of information about the proportion of exosomes and microvesicles in the collected “exosome” samples. This is understandable. From the perspective of exosome-based diagnosis and basic investigations, based on enriched exosome samples, rational experimental controls and optimized statistical models, as long as the composition of the studied samples could be accurately determined, the reliability of the assays could be ensured. For example, in functional studies conducted via siRNA-based gene regulation, it is unlikely, and not necessary, to completely inhibit the gene of interest. In general, an inhibition rate of around 80% is considered sufficient for most subsequent investigations. From the perspective of exosome-based drug development, according to the current drug approval system of most countries (including the United States, the European Union, Australia, China, and Japan), unlike the high purity required for chemical compounds, the requirements for quality control and safety assessment of cell-derived compounds such as exosomes can be met as soon as (1) the exosome proportion is sufficiently high and (2) individual components of the preparation can be quantified and described 11.

Therefore, the key question of current exosome studies is how to effectively quantify the individual components of the collected exosome samples. However, we cannot achieve this goal by solely relying on exosome markers, as reflected in various immunoaffinity-based commercial exosome extraction reagents (using antibodies targeting exosome markers). Although they claim to be able to enrich exosomes, some crucial information, including the proportion (or purity) of exosomes in the extract, and the content of other components (e.g., microvesicle), is invariably absent. Surely, even if a perfect exosome marker with 100% specificity is available for exosome isolation (this would be very unlikely in practice), it still cannot guarantee that the remaining vesicles are all microvesicles, which may contain excess exosomes, apoptotic bodies or protein precipitates. Moreover, in the absence of understanding of the microvesicle, we run into issues in determining the specificity of the employed exosome marker.

### 3.3 Combined application of exosome and microvesicle markers for quantitative exosome/microvesicle separation

**Figure 14:**
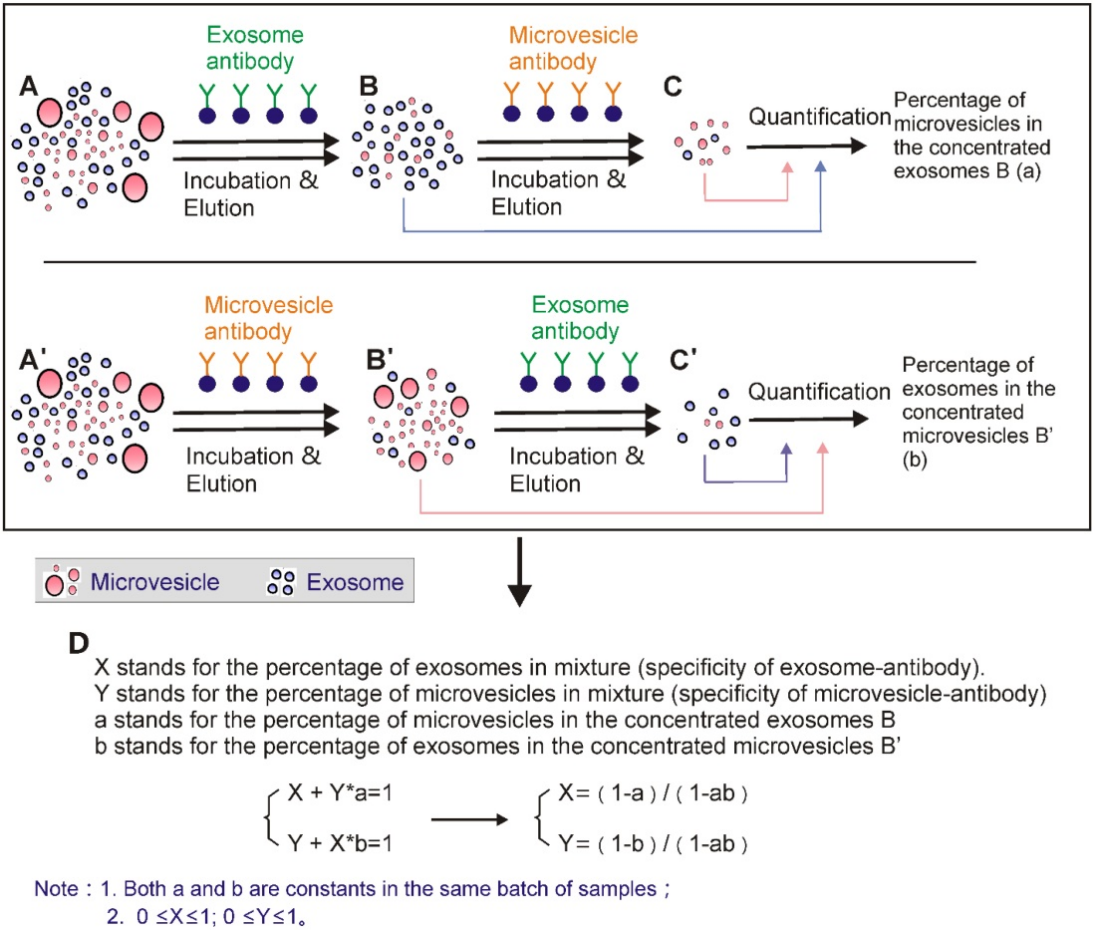
Calculation of the proportion of exosome and microvesicle components of extracellular vesicles via exosome and microvesicle marker-specific antibodies. The extracellular vesicles A (A') were concentrated via polymer precipitation. Then exosomes (B) or microvesicles (B') were extracted using corresponding antibody-based immunoaffinity capture; after elution, exosomes (C') and microvesicles (C) were extracted again from the elutes using antibody-based immunoaffinity method. Then, the extracted exosomes (B, C') and microvesicles (B', C) were quantified. The proportion of exosome and microvesicle in the original extracellular vesicles was calculated using the formulation as shown in D.

Since relying on biomarkers of only one component (i.e., exosome) cannot yield reliable quantitative information of individual components in the extracellular vesicle mixture, it seems that the only feasible method is to simultaneously employ both exosome and microvesicle markers. In fact, even if the applied biomarkers for exosome and microvesicle are not 100% specific, the respective markers of the two components combined with mathematical calculation (by Linear Equation in Two Unknowns, Figure 14) may present a reliable system to assess the proportion of exosomes and microvesicles. At the same time, by providing enriched microvesicles with quantitative information, the proposed separation system also provides opportunities for the investigation of microvesicle-based basic and clinical translations.

Having specific biological functions 147, 148, microvesicles must have stable protein expression. In recent years, although several microvesicle markers such as annexin A1 149, CD29 150, and Sca1 151 have been reported, bona fide markers for microvesicle separation are still not available 152. Future developments in specific microvesicle marker identification would promote quantitative exosome and microvesicle separation.

## 4. Perspective

Over the past few decades, despite the dramatic advances made in deciphering the mysteries of exosomes, the challenges in efficient exosome isolation have yet to be solved. This largely owes to the complexity of biological fluids, the considerable overlap of the physicochemical and biochemical properties among the exosomes, lipoproteins, virus, and other extracellular vesicles, as well as the heterogeneity of exosomes themselves 35. As a result, no specific exosome separation technique has currently been accepted as suitable for all studies 153. Depending on the biology samples applied, even the gold standard ultracentrifugation method often suffers from protein and lipoprotein contaminants. Under these circumstances, the combined application of two or more techniques presents a plausible strategy for efficient exosome isolation, as demonstrated by the previously reported combined use of immunoaffinity-based exosome capture (or ultrafiltration) and density-gradient centrifugation 96, 154, 155.

However, it should be noted that although combined isolation techniques result in higher exosome purity, they often increase procedure cost and complexity, thus resulting in reduced overall yield and unreliable downstream analysis. Therefore, the nature of the samples as well as the purpose of the investigation needs to be carefully considered when a particular combination of techniques is selected. For example, when the immunoaffinity capture method was used to process large volume samples, a pre-treatment via polymer precipitation 156 may be beneficial, to both promote the efficacy of the antibody-based exosome separation and avoid using excessive quantities of expensive antibodies.

For both diagnosis and therapeutic applications, researchers should carefully consider the strengths and weaknesses of the accessible strategies. As discussed previously, immunoaffinity capture promises selective isolation of highly purified exosomes of specific origins, or even subpopulations of exosomes from biological fluids. Therefore, in cases where diagnosis is scheduled for the subsequent investigations, immunoaffinity may present the most sensitive and specific method. Unfortunately, the current immunoaffinity method is compromised heavily by the lack of reliable markers for exosome isolation 17. Furthermore, when considering the heterogeneity in antigen expression, especially in cancer cells, the possibility of underestimation and false negatives must be noted 153. Even so, with the identification of more disease-specific exosome markers, coupled with recently developed microfabrication technologies (e.g., microfluidic), immunoaffinity-based exosome isolation may contribute greatly to future diseases diagnosis, especially through non-invasive liquid biopsy. Furthermore, as discussed in **Section 3,** isolation of specific exosome markers (as well as microvesicle markers) is also of great value to address the long-standing issue of quantitative exosome/microvesicle separation.

On the other hand, the therapeutic applications of exosomes are limited by the lack of an effective method to isolate high-quality exosomes in bulk 157, 158. In all likelihood, the ultrafiltration method may contribute most to this area due to its advantageous features, including ease of handling and analyzing large batches of biological samples, and capability of isolating exosomes with high purity and defined sizes. However, despite increasing popularity, ultrafiltration is not without its limitations, especially the problem of membrane clogging and vesicle trapping. This results in not only reduced lifetime of the expensive membranes, but also reduced isolation efficiency and erroneous interpretations of test results. Fortunately, this issue can be addressed by tangential flow filtration. Although current tangential flow filtration techniques are still limited by processing volume, given the ongoing progress in hydromechanics and material sciences, we believe the isolation efficiency of future ultrafiltration methods will be dramatically improved. Similarly, SEC, which features both high-quality exosome preparation and excellent reproducibility, also holds great potential for high-throughput industrial applications. This is especially true given the fact that the gravity flow used in SEC causes minimal damage to exosome structure and function. Collectively, we reckon that ultrafiltration and SEC may provide a basis for future standardization of clinical grade exosome samples.

Conversely, no microfluidic device has been readily applied in clinical applications, in spite of the remarkable advances achieved in recent years. Major roadblocks to clinical applications include standardization, scalability, and validation 127, 159. Furthermore, the relatively low isolation efficiency of such methods may pose detrimental effects on downstream assessments such as genomic and proteomic analysis, and result in compromised diagnosis results. We believe further improvements in microfluidic processing capacity via multiple exosome sorting mechanisms, as well as massively parallel microfluidic sets, represents plausible solutions. Importantly, most of the existing exosome isolation techniques are applied to basic research. For microfluidics to become more clinically relevant we suggest the design of techniques and devices for exosome isolation should take a more translational approach, by thoroughly evaluating a sufficient number of clinical samples for improved selectivity, robustness, and sensitivity.

Furthermore, to facilitate the in-depth investigation of exosomes and their related biological functions, more efforts need to be made for the development of simultaneous exosome separation/quantification strategies and devices, to achieve not only efficient exosome isolation, but also real-time exosome quantification and analysis. Although several real-time exosome isolation/detection apparatuses have been reported, standardizing the analysis module for comparable and reliable readouts still represents a great challenge. In fact, compared to analysis, development of standardized exosome isolation methods constitutes an even harder task. Due to the heterogeneous features of biological samples, a reliable exosome separation technique suitable for every study is still not available. A bespoke selection of separation methods tailored for particular exosome-containing objects is imperative for high-quality exosome isolation and readout validation. As a result, future efforts may need to develop different exosome isolation standards to meet the particular properties of different types of biological samples and target particles (i.e., genetic or protein contents) to be screened.

## 5. Conclusion

The observation of exosome-mediated cell signaling provides a great opportunity for developing exosome-related basic and applied biomedical applications in various fields. Despite the revolutionary progress in exosome-based theranostic applications over the past few decades, there are still fundamental unanswered questions in the field. These questions address some of the hotspots of current biomedical research such as the secretory regulation mechanism of exosomes, exosomal content sorting mechanism and their intercellular transduction pathway. Various exosome separation strategies and devices have been suggested to facilitate the investigation of exosomes and their related biological functions. As comprehensively discussed in this work, standardization in exosome preparation such as specimen handling, isolation, and quantification has still not been established. Through studying the nature of particular samples and specific application settings, we believe careful selection of isolation techniques (or a combination of isolation techniques) will help investigators address many of the challenges faced in current exosome studies. In addition, we also believe that the exosome/microvesicle separation and quantification strategy (using both exosome and microvesicle markers) as suggested in this work can provide a plausible strategy to obtain accurate quantitative information for future exosome (and microvesicle)-related investigations.

## Figures and Tables

**Table 1:** Current strategies for exosome separation

Isolation technique	Principle	Advantages	Disadvantages
Sequential ultracentrifugation	Particles have different density and size show different sediment speed under centrifugal force	• Low cost and • Low contamination risk with extra isolation reagents; •suitable for large volume preparation;	• High equipment requirement • Time consuming • Labor intensive • Potential mechanical damage due to high speed centrifugation • Protein aggregation • Not suitable for small volume diagnosis • Low portability
Gradient ultracentrifugation	After centrifugation in a dense medium, objects in a tube could stay in the position of the medium with similar density	• High purity of products • Allowing separation of subpopulation of exosomes	• Lower volume processability • High equipment requirement • Time consuming • Labor intensive • Potential mechanical damage due to high speed centrifugation • Not suitable for small volume diagnosis • Low portability
Ultrafiltration	Utilizing filter membrane with defined size-exclusion limit or molecular weight cut-off	• Low equipment cost • Fast procedure • good portability	• Moderate purity • Potential deterioration induced by shear stress • Possible loss due to clogging and membrane trapping
Size-exclusion chromatography	After adding to porous materials, substances eluted out in accordance with their particle size, with big particles eluted earlier	• High purity • Fast preparation • Keep native state of exosomes • Good reproducibility • Potential for both small and large sample capacity; • Capable of processing all type of samples	• Relatively high device costs • Additional method for exosome enrichment is required
Polymer Precipitation	High hydrophilic water-excluding polymers can alternate the solubility of exosomes	• Easy to use • Using ordinary equipment •Suitable for both small and large sample volume • High efficiency	• Contaminants of protein aggregates, other extracellular vesicles and polymeric contaminants • Extended processing time • Require complicated clean-up steps • Affecting downstream analysis and quantification
Immunoaffinity capture	Based on specific binding between exosome markers and immobilized antibodies (ligands)	• Suitable for separating exosomes of specific origin; • High-purity exosomes • Easy to use • No chemical contamination	• High-cost antibodies; • Exosome markers must be optimized • Low processing volume and yields • Extra step for exosome elution may damage native exosome structure
Microfluidics-based techniques	Based on different principles including immunoaffinity, size and density	• Highly efficient • Cost-effective • Portable • Easily automated & integrated with diagnosis	• Low sample capacity

## References

[1] Pickett J (2007). Cell signalling - New communication skills. Nat Rev Mol Cell Bio.

[2] Farooqi AA, Desai NN, Qureshi MZ, Librelotto DRN, Gasparri ML, Bishayee A (2018). Exosome biogenesis, bioactivities and functions as new delivery systems of natural compounds. Biotechnol Adv.

[3] Johnstone RM, Adam M, Hammond JR, Orr L, Turbide C (1987). Vesicle Formation during Reticulocyte Maturation - Association of Plasma-Membrane Activities with Released Vesicles (Exosomes). J Biol Chem.

[4] Valadi H, Ekstrom K, Bossios A, Sjostrand M, Lee JJ, Lotvall JO (2007). Exosome-mediated transfer of mRNAs and microRNAs is a novel mechanism of genetic exchange between cells. Nat Cell Biol.

[5] Kilchert C, Wittmann S, Vasiljeva L (2016). The regulation and functions of the nuclear RNA exosome complex. Nat Rev Mol Cell Bio.

[6] Tran PHL, Wang T, Yin W, Tran TTD, Barua HT, Zhang YM (2019). Development of a nanoamorphous exosomal delivery system as an effective biological platform for improved encapsulation of hydrophobic drugs. Int J Pharmaceut.

[7] Barros FM, Carneiro F, Machado JC, Melo SA (2018). Exosomes and immune response in cancer: friends or foes?. Front Immunol.

[8] Zhao T, Sun F, Liu J, Ding T, She J, Mao F (2019). Emerging Role of Mesenchymal Stem Cell-derived Exosomes in Regenerative Medicine. Curr Stem Cell Res Ther.

[9] Lener T, Gimona M, Aigner L, Borger V, Buzas E, Camussi G (2015). Applying extracellular vesicles based therapeutics in clinical trials - an ISEV position paper. J Extracell Vesicles.

[10] Tcherpakov M. Exosome Diagnostics and Therapeutics: Global Markets.; 2018

[11] Fais S, O'Driscoll L, Borras FE, Buzas E, Camussi G, Cappello F (2016). Evidence-based clinical use of nanoscale extracellular vesicles in nanomedicine. Acs Nano.

[12] Willms E, Cabanas C, Mager I, Wood MJA, Vader P (2018). Extracellular vesicle heterogeneity: subpopulations, isolation techniques, and diverse functions in cancer progression. Front Immunol.

[13] Li P, Kaslan M, Lee SH, Yao J, Gao Z (2017). Progress in exosome isolation techniques. Theranostics.

[14] Karimi N, Cvjetkovic A, Jang SC, Crescitelli R, Feizi MAH, Nieuwland R (2018). Detailed analysis of the plasma extracellular vesicle proteome after separation from lipoproteins. Cell Mol Life Sci.

[15] Thery C, Witwer KW, Aikawa E, Alcaraz MJ, Anderson JD, Andriantsitohaina R (2018). Minimal information for studies of extracellular vesicles 2018 (MISEV2018): a position statement of the International Society for Extracellular Vesicles and update of the MISEV2014 guidelines. J Extracell Vesicles.

[16] Johnstone RM, Bianchini A, Teng K (1989). Reticulocyte maturation and exosome release: transferrin receptor containing exosomes shows multiple plasma membrane functions. Blood.

[17] Konoshenko MY, Lekchnov EA, Vlassov AV, Laktionov PP (2018). Isolation of extracellular vesicles: general methodologies and latest trends. Biomed Res Int.

[18] He L, Zhu W, Chen Q, Yuan Y, Wang Y, Wang J (2019). Ovarian cancer cell-secreted exosomal miR-205 promotes metastasis by inducing angiogenesis. Theranostics.

[19] Thery C, Amigorena S, Raposo G, Clayton A (2006). Isolation and characterization of exosomes from cell culture supernatants and biological fluids.

[20] Muller L, Hong CS, Stolz DB, Watkins SC, Whiteside TL (2014). Isolation of biologically-active exosomes from human plasma. J Immunol Methods.

[21] Hiemstra TF, Charles PD, Gracia T, Hester SS, Gatto L, Al-Lamki R (2014). Human urinary exosomes as innate immune effectors. J Am Soc Nephrol.

[22] Livshits MA, Khomyakova E, Evtushenko EG, Lazarev VN, Kulemin NA, Semina SE (2016). Isolation of exosomes by differential centrifugation: Theoretical analysis of a commonly used protocol (vol 5, 17319, 2015).

[23] Langevin SM, Kuhnell D, Orr-Asman MA, Biesiada J, Zhang X, Medvedovic M (2019). Balancing yield, purity and practicality: a modified differential ultracentrifugation protocol for efficient isolation of small extracellular vesicles from human serum. RNA Biol.

[24] Chia BS, Low YP, Wang Q, Li P, Gao ZQ (2017). Advances in exosome quantification techniques. Trac-Trend Anal Chem.

[25] Paolini L, Zendrini A, Di Noto G, Busatto S, Lottini E, Radeghieri A (2016). Residual matrix from different separation techniques impacts exosome biological activity. Sci Rep.

[26] Zhu JM, Liu B, Wang ZY, Wang D, Ni HE, Zhang LL (2019). Exosomes from nicotine-stimulated macrophages accelerate atherosclerosis through miR-21-3p/PTEN-mediated VSMC migration and proliferation. Theranostics.

[27] Gao L, Mei S, Zhang S, Qin Q, Li H, Liao Y (2020). Cardio-renal exosomes in myocardial infarction serum regulate proangiogenic paracrine signaling in adipose mesenchymal stem cells. Theranostics.

[28] Schuldner M, Dorsam B, Shatnyeva O, Reiners KS, Kubarenko A, Hansen HP (2019). Exosome-dependent immune surveillance at the metastatic niche requires BAG6 and CBP/p300-dependent acetylation of p53. Theranostics.

[29] Brakke MK (1951). Density gradient centrifugation - a new separation technique. J Am Chem Soc.

[30] Kim B, Kim KH, Chang Y, Shin S, Shin EC, Choi S (2019). One-step microfluidic purification of white blood cells from whole blood for immunophenotyping.

[31] Perez-Gonzalez R, Gauthier SA, Kumar A, Saito M, Saito M, Levy E (2017). A method for isolation of extracellular vesicles and characterization of exosomes from brain extracellular space. Methods Mol Biol.

[32] Street JM, Koritzinsky EH, Glispie DM, Yuen PST (2017). Urine exosome isolation and characterization. Methods Mol Biol.

[33] Tran PHL, Wang T, Yin W, Tran TTD, Nguyen TNG, Lee BJ (2019). Aspirin-loaded nanoexosomes as cancer therapeutics.

[34] Onodi Z, Pelyhe C, Terezia Nagy C, Brenner GB, Almasi L, Kittel A (2018). Isolation of high-purity extracellular vesicles by the combination of iodixanol density gradient ultracentrifugation and bind-elute chromatography from blood plasma. Front Physiol.

[35] Chen BY, Sung CW, Chen C, Cheng CM, Lin DP, Huang CT (2019). Advances in exosomes technology. Clin Chim Acta.

[36] Lim YJ, Lee SJ (2017). Are exosomes the vehicle for protein aggregate propagation in neurodegenerative diseases?. Acta Neuropathol Commun.

[37] Van Deun J, Mestdagh P, Sormunen R, Cocquyt V, Vermaelen K, Vandesompele J (2014). The impact of disparate isolation methods for extracellular vesicles on downstream RNA profiling.

[38] He L, Zhu D, Wang J, Wu X (2019). A highly efficient method for isolating urinary exosomes. Int J Mol Med.

[39] Yu LL, Zhu J, Liu JX, Jiang F, Ni WK, Qu LS (2018). A comparison of traditional and novel methods for the separation of exosomes from human samples. Biomed Res Int.

[40] Heinemann ML, Vykoukal J (2017). Sequential filtration: a gentle method for the isolation of functional extracellular vesicles. Methods Mol Biol.

[41] Dehghani M, Lucas K, Flax J, McGrath J, Gaborski T (2019). Tangential flow microfluidics for the capture and release of nanoparticles and extracellular vesicles on conventional and ultrathin membranes.

[42] Popovic M, de Marco A (2018). Canonical and selective approaches in exosome purification and their implications for diagnostic accuracy. Transl Cancer Res.

[43] Doyle LM, Wang MZ (2019). Overview of Extracellular Vesicles, Their Origin, Composition, Purpose, and Methods for Exosome Isolation and Analysis.

[44] Soda N, Rehm BHA, Sonar P, Nguyen NT, Shiddiky MJA (2019). Advanced liquid biopsy technologies for circulating biomarker detection. J Mater Chem B.

[45] Peterson MF, Otoc N, Sethi JK, Gupta A, Antes TJ (2015). Integrated systems for exosome investigation. Methods.

[46] Martins TS, Catita J, Rosa IM, Silva OABDE, Henriques AG (2018). Exosome isolation from distinct biofluids using precipitation and column-based approaches.

[47] Gheinani AH, Vogeli M, Baumgartner U, Vassella E, Draeger A, Burkhard FC (2018). Improved isolation strategies to increase the yield and purity of human urinary exosomes for biomarker discovery.

[48] Musumeci T, Leonardi A, Bonaccorso A, Pignatello R, Puglisi G (2018). Tangential flow filtration technique: an overview on nanomedicine applications. Pharm Nanotechnol.

[49] Elmer J, Harris D, Palmer AF (2011). Purification of hemoglobin from red blood cells using tangential flow filtration and immobilized metal ion affinity chromatography. J Chromatogr B Analyt Technol Biomed Life Sci.

[50] Lebreton B, Brown A, van Reis R (2008). Application of high-performance tangential flow filtration (HPTFF) to the purification of a human pharmaceutical antibody fragment expressed in Escherichia coli. Biotechnol Bioeng.

[51] Escudier B, Dorval T, Chaput N, Andre F, Caby MP, Novault S (2005). Vaccination of metastatic melanoma patients with autologous dendritic cell (DC) derived-exosomes: results of thefirst phase I clinical trial. J Transl Med.

[52] Morse MA, Garst J, Osada T, Khan S, Hobeika A, Clay TM (2005). A phase I study of dexosome immunotherapy in patients with advanced non-small cell lung cancer. J Transl Med.

[53] Besse B, Charrier M, Lapierre V, Dansin E, Lantz O, Planchard D (2016). Dendritic cell-derived exosomes as maintenance immunotherapy after first line chemotherapy in NSCLC.

[54] Alvarez ML, Khosroheidari M, Ravi RK, DiStefano JK (2012). Comparison of protein, microRNA, and mRNA yields using different methods of urinary exosome isolation for the discovery of kidney disease biomarkers. Kidney Int.

[55] Patel GK, Khan MA, Zubair H, Srivastava SK, Khushman M, Singh S (2019). Comparative analysis of exosome isolation methods using culture supernatant for optimum yield, purity and downstream applications. Sci Rep.

[56] Lobb RJ, Becker M, Wen SW, Wong CS, Wiegmans AP, Leimgruber A (2015). Optimized exosome isolation protocol for cell culture supernatant and human plasma. J Extracell Vesicles.

[57] Cheruvanky A, Zhou H, Pisitkun T, Kopp JB, Knepper MA, Yuen PST (2007). Rapid isolation of urinary exosomal biomarkers using a nanomembrane ultrafiltration concentrator. Am J Physiol-Renal.

[58] Taylor DD, Shah S (2015). Methods of isolating extracellular vesicles impact down-stream analyses of their cargoes. Methods.

[59] Lathe GH, Ruthven CR (1955). The separation of substances on the basis of their molecular weights, using columns of starch and water. Biochem J.

[60] Ruysschaert T, Marque A, Duteyrat JL, Lesieur S, Winterhalter M, Fournier D (2005). Liposome retention in size exclusion chromatography. BMC Biotechnol.

[61] Brezinski K, Gorczyca B (2019). An overview of the uses of high performance size exclusion chromatography (HPSEC) in the characterization of natural organic matter (NOM) in potable water, and ion-exchange applications. Chemosphere.

[62] Hong P, Koza S, Bouvier ESP (2012). A review size-exclusion chromatography for the analysis of protein biotherapeutics and their aggregates. J Liq Chromatogr R T.

[63] Gamez-Valero A, Monguio-Tortajada M, Carreras-Planella L, Franquesa M, Beyer K, Borras FE (2016). Size-exclusion chromatography-based isolation minimally alters extracellular vesicles' characteristics compared to precipitating agents. Sci Rep.

[64] An MR, Wu J, Zhu JH, Lubman DM (2018). Comparison of an optimized ultracentrifugation method versus size-exclusion chromatography for isolation of exosomes from human serum. J Proteome Res.

[65] Stranska R, Gysbrechts L, Wouters J, Vermeersch P, Bloch K, Dierickx D (2018). Comparison of membrane affinity-based method with size-exclusion chromatography for isolation of exosome-like vesicles from human plasma.

[66] Navajas R, Corrales FJ, Paradela A (2019). Serum exosome isolation by size-exclusion chromatography for the discovery and validation of preeclampsia-associated biomarkers. Methods Mol Biol.

[67] Ma C, Jiang F, Ma Y, Wang J, Li H, Zhang J (2019). Isolation and Detection Technologies of Extracellular Vesicles and Application on Cancer Diagnostic. Dose Response.

[68] Witwer KW, Buzas EI, Bemis LT, Bora A, Lasser C, Lotvall J (2013). Standardization of sample collection, isolation and analysis methods in extracellular vesicle research.

[69] Shu S, Yang Y, Allen CL, Hurley E, Tung KH, Minderman H (2020). Purity and yield of melanoma exosomes are dependent on isolation method. J Extracell Vesicles.

[70] Rood IM, Deegens JKJ, Merchant ML, Tamboer WPM, Wilkey DW, Wetzels JFM (2010). Comparison of three methods for isolation of urinary microvesicles to identify biomarkers of nephrotic syndrome. Kidney Int.

[71] Garcia-Romero N, Madurga R, Rackov G, Palacin-Aliana I, Nunez-Torres R, Asensi-Puig A (2019). Polyethylene glycol improves current methods for circulating extracellular vesicle-derived DNA isolation. Transl Med Commun.

[72] Dou YQ, Kong P, Li CL, Sun HX, Li WW, Yu Y (2020). Smooth muscle SIRT1 reprograms endothelial cells to suppress angiogenesis after ischemia. Theranostics.

[73] Kanchi Ravi R, Khosroheidari M, DiStefano JK (2015). A modified precipitation method to isolate urinary exosomes.

[74] Wan Z, Zhao LB, Lu F, Gao XT, Dong Y, Zhao YX (2020). Mononuclear phagocyte system blockade improves therapeutic exosome delivery to the myocardium. Theranostics.

[75] Merdalimova A, Chernyshev V, Nozdriukhin D, Rudakovskaya P, Gorin D, Yashchenok A (2019). Identification and analysis of exosomes by surface-enhanced raman spectroscopy.

[76] Sim SL, He T, Tscheliessnig A, Mueller M, Tan RB, Jungbauer A (2012). Protein precipitation by polyethylene glycol: a generalized model based on hydrodynamic radius. J Biotechnol.

[77] Kimura T, Ferran B, Tsukahara Y, Shang Q, Desai S, Fedoce A (2019). Production of adeno-associated virus vectors for in vitro and in vivo applications. Sci Rep.

[78] Gyorgy B, Modos K, Pallinger E, Paloczi K, Pasztoi M, Misjak P (2011). Detection and isolation of cell-derived microparticles are compromised by protein complexes resulting from shared biophysical parameters. Blood.

[79] Kalra H, Adda CG, Liem M, Ang CS, Mechler A, Simpson RJ (2013). Comparative proteomics evaluation of plasma exosome isolation techniques and assessment of the stability of exosomes in normal human blood plasma. Proteomics.

[80] Hartjes TA, Mytnyk S, Jenster GW, van Steijn V, van Royen ME (2019). Extracellular vesicle quantification and characterization: common methods and emerging approaches.

[81] Kirbas OK, Bozkurt BT, Asutay AB, Mat B, Ozdemir B, Ozturkoglu D (2019). Optimized isolation of extracellular vesicles from various organic sources using aqueous two-phase system.

[82] Iqbal M, Tao Y, Xie S, Zhu Y, Chen D, Wang X (2016). Aqueous two-phase system (ATPS): an overview and advances in its applications. Biol Proced Online.

[83] Kastelowitz N, Yin H (2014). Exosomes and microvesicles: identification and targeting by particle size and lipid chemical probes. Chembiochem.

[84] Zhang Y, Liu YF, Liu HY, Tang WH (2019). Exosomes: biogenesis, biologic function and clinical potential.

[85] Smolarz M, Pietrowska M, Matysiak N, Mielanczyk L, Widlak P (2019). Proteome profiling of exosomes purified from a small amount of human serum: the problem of co-purified serum components.

[86] Zara M, Guidetti GF, Camera M, Canobbio I, Amadio P, Torti M (2019). Biology and role of extracellular vesicles (evs) in the pathogenesis of thrombosis.

[87] Brzozowski JS, Jankowski H, Bond DR, McCague SB, Munro BR, Predebon MJ (2018). Lipidomic profiling of extracellular vesicles derived from prostate and prostate cancer cell lines.

[88] Andreu Z, Yanez-Mo M (2014). Tetraspanins in extracellular vesicle formation and function. Front Immunol.

[89] Liu C, Su C (2019). Design strategies and application progress of therapeutic exosomes. Theranostics.

[90] Rupp AK, Rupp C, Keller S, Brase JC, Ehehalt R, Fogel M (2011). Loss of EpCAM expression in breast cancer derived serum exosomes: role of proteolytic cleavage. Gynecol Oncol.

[91] Huang T, Deng CX (2019). Current progresses of exosomes as cancer diagnostic and prognostic biomarkers. Int J Biol Sci.

[92] Zarovni N, Corrado A, Guazzi P, Zocco D, Lari E, Radano G (2015). Integrated isolation and quantitative analysis of exosome shuttled proteins and nucleic acids using immunocapture approaches. Methods.

[93] Xian PP, Hei Y, Wang R, Wang T, Yang JL, Li JY (2019). Mesenchymal stem cell-derived exosomes as a nanotherapeutic agent for amelioration of inflammation-induced astrocyte alterations in mice. Theranostics.

[94] Nakai W, Yoshida T, Diez D, Miyatake Y, Nishibu T, Imawaka N (2016). A novel affinity-based method for the isolation of highly purified extracellular vesicles. Sci Rep.

[95] Yoshida T, Ishidome T, Hanayama R (2017). High purity isolation and sensitive quantification of extracellular vesicles using affinity to TIM4. Curr Protoc Cell Biol.

[96] Kang YT, Kim YJ, Bu J, Cho YH, Han SW, Moon BI (2017). High-purity capture and release of circulating exosomes using an exosome-specific dual-patterned immunofiltration (ExoDIF) device. Nanoscale.

[97] Guo SC, Tao SC, Dawn H (2018). Microfluidics-based on-a-chip systems for isolating and analysing extracellular vesicles. J Extracell Vesicles.

[98] Wang T, Shigdar S, Gantier MP, Hou Y, Wang L, Li Y (2015). Cancer stem cell targeted therapy: progress amid controversies. Oncotarget.

[99] Conde-Vancells J, Rodriguez-Suarez E, Embade N, Gil D, Matthiesen R, Valle M (2008). Characterization and comprehensive proteome profiling of exosomes secreted by hepatocytes. J Proteome Res.

[100] Samsonov R, Shtam T, Burdakov V, Glotov A, Tsyrlina E, Berstein L (2016). Lectin-induced agglutination method of urinary exosomes isolation followed by mi-RNA analysis: application for prostate cancer diagnostic. Prostate.

[101] Balaj L, Atai NA, Chen W, Mu D, Tannous BA, Breakefield XO (2015). Heparin affinity purification of extracellular vesicles. Sci Rep.

[102] He C, Zheng S, Luo Y, Wang B (2018). Exosome theranostics: biology and translational medicine. Theranostics.

[103] Shigdar S, Qiao L, Zhou SF, Xiang D, Wang T, Li Y (2013). RNA aptamers targeting cancer stem cell marker CD133. Cancer Lett.

[104] Wang T, Gantier MP, Xiang D, Bean AG, Bruce M, Zhou SF (2015). EpCAM Aptamer-mediated Survivin Silencing Sensitized Cancer Stem Cells to Doxorubicin in a Breast Cancer Model. Theranostics.

[105] Wang T, Yin W, AlShamaileh H, Zhang Y, Tran PH, Nguyen TN (2019). A detailed protein-SELEX protocol allowing visual assessments of individual steps for a high success rate. Hum Gene Ther Methods.

[106] Wang T, Philippovich S, Mao J, Veedu RN (2019). efficient epidermal growth factor receptor targeting oligonucleotide as a potential molecule for targeted cancer therapy.

[107] Zhang K, Yue Y, Wu S, Liu W, Shi J, Zhang Z (2019). Rapid capture and nondestructive release of extracellular vesicles using aptamer-based magnetic isolation. ACS Sens.

[108] Yu X, He L, Pentok M, Yang H, Yang Y, Li Z (2019). An aptamer-based new method for competitive fluorescence detection of exosomes. Nanoscale.

[109] Wang T, Chen CY, Larcher LM, Barrero RA, Veedu RN (2019). Three decades of nucleic acid aptamer technologies: Lessons learned, progress and opportunities on aptamer development. Biotechnol Adv.

[110] Wang T, Rahimizadeh K, Veedu RN (2019). Development of a novel dna oligonucleotide targeting low-density lipoprotein receptor. Mol Ther Nucleic Acids.

[111] Salieb-Beugelaar GB, Simone G, Arora A, Philippi A, Manz A (2010). Latest developments in microfluidic cell biology and analysis systems. Anal Chem.

[112] Jackson EL, Lu H (2013). Advances in microfluidic cell separation and manipulation. Curr Opin Chem Eng.

[113] Gholizadeh S, Shehata Draz M, Zarghooni M, Sanati-Nezhad A, Ghavami S, Shafiee H (2017). Microfluidic approaches for isolation, detection, and characterization of extracellular vesicles: Current status and future directions. Biosens Bioelectron.

[114] Contreras-Naranjo JC, Wu HJ, Ugaz VM (2017). Microfluidics for exosome isolation and analysis: enabling liquid biopsy for personalized medicine. Lab Chip.

[115] Zhang P, Zhou X, He M, Shang Y, Tetlow AL, Godwin AK (2019). Ultrasensitive detection of circulating exosomes with a 3D-nanopatterned microfluidic chip. Nat Biomed Eng.

[116] Kanwar SS, Dunlay CJ, Simeone DM, Nagrath S (2014). Microfluidic device (ExoChip) for on-chip isolation, quantification and characterization of circulating exosomes. Lab Chip.

[117] Chen C, Skog J, Hsu CH, Lessard RT, Balaj L, Wurdinger T (2010). Microfluidic isolation and transcriptome analysis of serum microvesicles. Lab Chip.

[118] Zhang P, He M, Zeng Y (2016). Ultrasensitive microfluidic analysis of circulating exosomes using a nanostructured graphene oxide/polydopamine coating. Lab Chip.

[119] Hisey CL, Dorayappan KDP, Cohn DE, Selvendiran K, Hansford DJ (2018). Microfluidic affinity separation chip for selective capture and release of label-free ovarian cancer exosomes. Lab Chip.

[120] Vaidyanathan R, Naghibosadat M, Rauf S, Korbie D, Carrascosa LG, Shiddiky MJA (2014). Detecting exosomes specifically: a multiplexed device based on alternating current electrohydrodynamic induced nanoshearing. Anal chem.

[121] Dudani JS, Gossett DR, Tse HTK, Lamm RJ, Kulkarni RP, Di Carlo D (2015). Rapid inertial solution exchange for enrichment and flow cytometric detection of microvesicles.

[122] Bairen Pang YZ, Jie Ni, James Thompson, David Malouf, Joseph Bucci, Peter Graham, Yong Li (2020). Extracellular vesicles: the next generation of biomarkers for liquid biopsy-based prostate cancer diagnosis. Theranostics.

[123] Wang ZX, Wu HJ, Fine D, Schmulen J, Hu Y, Godin B (2013). Ciliated micropillars for the microfluidic-based isolation of nanoscale lipid vesicles. Lab Chip.

[124] Yuan D, Zhao QB, Yan S, Tang SY, Alici G, Zhang J (2018). Recent progress of particle migration in viscoelastic fluids. Lab Chip.

[125] Liu C, Guo JY, Tian F, Yang N, Yan FS, Ding YP (2017). Field-free isolation of exosomes from extracellular vesicles by microfluidic viscoelastic flows. Acs Nano.

[126] Chiriaco MS, Bianco M, Nigro A, Primiceri E, Ferrara F, Romano A (2018). Lab-on-chip for exosomes and microvesicles detection and characterization.

[127] Iliescu FS, Vrtacnik D, Neuzil P, Iliescu C (2019). Microfluidic technology for clinical applications of exosomes.

[128] Bruus H (2012). Acoustofluidics 7: The acoustic radiation force on small particles. Lab Chip.

[129] Lee K, Shao HL, Weissleder R, Lee H (2015). Acoustic purification of extracellular microvesicles. Acs Nano.

[130] Wu M, Ouyang Y, Wang Z, Zhang R, Huang PH, Chen C (2017). Isolation of exosomes from whole blood by integrating acoustics and microfluidics. Proc Natl Acad Sci U S A.

[131] Ayala-Mar S, Gallo-Villanueva RC, Gonzalez-Valdez J (2019). Dielectrophoretic manipulation of exosomes in a multi-section microfluidic device. Mater Today Proc.

[132] Davies RT, Kim J, Jang SC, Choi EJ, Gho YS, Park J (2012). Microfluidic filtration system to isolate extracellular vesicles from blood. Lab Chip.

[133] Shi LL, Rana A, Esfandiari L (2018). A low voltage nanopipette dielectrophoretic device for rapid entrapment of nanoparticles and exosomes extracted from plasma of healthy donors.

[134] Ayala-Mar S, Perez-Gonzalez VH, Mata-Gomez MA, Gallo-Villanueva RC, Gonzalez-Valdez J (2019). Electrokinetically driven exosome separation and concentration using dielectrophoretic-enhanced pdms-based microfluidics. Anal Chem.

[135] Marczak S, Richards K, Ramshani Z, Smith E, Senapati S, Hill R (2018). Simultaneous isolation and preconcentration of exosomes by ion concentration polarization. Electrophoresis.

[136] Ashcroft BA, de Sonneville J, Yuana Y, Osanto S, Bertina R, Kuil ME (2012). Determination of the size distribution of blood microparticles directly in plasma using atomic force microscopy and microfluidics. Biomed Microdevices.

[137] He M, Crow J, Roth M, Zeng Y, Godwin AK (2014). Integrated immunoisolation and protein analysis of circulating exosomes using microfluidic technology. Lab Chip.

[138] Zhao Z, Yang Y, Zeng Y, He M (2016). A microfluidic ExoSearch chip for multiplexed exosome detection towards blood-based ovarian cancer diagnosis. Lab Chip.

[139] Ueda K, Ishikawa N, Tatsuguchi A, Saichi N, Fujii R, Nakagawa H (2014). Antibody-coupled monolithic silica microtips for highthroughput molecular profiling of circulating exosomes.

[140] Street JM, Barran PE, Mackay CL, Weidt S, Balmforth C, Walsh TS (2012). Identification and proteomic profiling of exosomes in human cerebrospinal fluid. J Transl Med.

[141] Guix FX, Corbett GT, Cha DJ, Mustapic M, Liu W, Mengel D (2018). Detection of aggregation-competent Tau in neuron-derived extracellular vesicles.

[142] Sedgwick AE, D'Souza-Schorey C (2018). The biology of extracellular microvesicles. Traffic.

[143] Al-Nedawi K, Meehan B, Micallef J, Lhotak V, May L, Guha A (2008). Intercellular transfer of the oncogenic receptor EGFRvIII by microvesicles derived from tumour cells. Nat Cell Biol.

[144] Wang T, Shigdar S, Shamaileh HA, Gantier MP, Yin W, Xiang D (2017). Challenges and opportunities for siRNA-based cancer treatment. Cancer Lett.

[145] Belov L, Matic KJ, Hallal S, Best OG, Mulligan SP, Christopherson RI (2016). Extensive surface protein profiles of extracellular vesicles from cancer cells may provide diagnostic signatures from blood samples. J Extracell Vesicles.

[146] Kowal J, Arras G, Colombo M, Jouve M, Morath JP, Primdal-Bengtson B (2016). Proteomic comparison defines novel markers to characterize heterogeneous populations of extracellular vesicle subtypes. Proc Natl Acad Sci U S A.

[147] Dionisi M, De Archangelis C, Battisti F, Koshkaki HR, Belleudi F, Zizzari IG (2018). Tumor-derived microvesicles enhance cross-processing ability of clinical grade dendritic cells.

[148] Zhang D, Lee H, Wang X, Groot M, Sharma L, Dela Cruz CS (2019). A potential role of microvesicle-containing miR-223/142 in lung inflammation. Thorax.

[149] Jeppesen DK, Fenix AM, Franklin JL, Higginbotham JN, Zhang Q, Zimmerman LJ (2019). Reassessment of exosome composition. Cell.

[150] Liu Z, Xu Y, Wan Y, Gao J, Chu Y, Li J (2019). Exosomes from adipose-derived mesenchymal stem cells prevent cardiomyocyte apoptosis induced by oxidative stress. Cell Death Discov.

[151] Borgovan T, Crawford L, Nwizu C, Quesenberry P (2019). Stem cells and extracellular vesicles: biological regulators of physiology and disease. Am J Physiol Cell Physiol.

[152] Lener T, Gimona M, Aigner L, Borger V, Buzas E, Camussi G (2015). Applying extracellular vesicles based therapeutics in clinical trials - an ISEV position paper. J Extracell Vesicles.

[153] Ludwig N, Whiteside TL, Reichert TE (2019). Challenges in exosome isolation and analysis in health and disease.

[154] Konadu KA, Huang MB, Roth W, Armstrong W, Powell M, Villinger F (2016). Isolation of exosomes from the plasma of HIV-1 positive individuals.

[155] Gao X, Wan Z, Wei M, Dong Y, Zhao Y, Chen X (2019). Chronic myelogenous leukemia cells remodel the bone marrow niche via exosome-mediated transfer of miR-320. Theranostics.

[156] Kornilov R, Puhka M, Mannerstrom B, Hiidenmaa H, Peltoniemi H, Siljander P (2018). Efficient ultrafiltration-based protocol to deplete extracellular vesicles from fetal bovine serum.

[157] Willis GR, Kourembanas S, Mitsialis SA (2017). Toward exosome-based therapeutics: isolation, heterogeneity, and fit-for-purpose potency. Front Cardiovasc Med.

[158] Murphy DE, de Jong OG, Brouwer M, Wood MJ, Lavieu G, Schiffelers RM (2019). Extracellular vesicle-based therapeutics: natural versus engineered targeting and trafficking. Exp Mol Med.

[159] Liga A, Vliegenthart AD, Oosthuyzen W, Dear JW, Kersaudy-Kerhoas M (2015). Exosome isolation: a microfluidic road-map. Lab Chip.

